# Ruthenium(II) Polypyridyl Complexes for Antimicrobial Photodynamic Therapy: Prospects for Application in Cystic Fibrosis Lung Airways

**DOI:** 10.3390/pharmaceutics14081664

**Published:** 2022-08-10

**Authors:** Raphaëlle Youf, Adeel Nasir, Mareike Müller, Franck Thétiot, Tanguy Haute, Rosy Ghanem, Ulrich Jonas, Holger Schönherr, Gilles Lemercier, Tristan Montier, Tony Le Gall

**Affiliations:** 1 INSERM, Univ Brest, EFS, UMR 1078, GGB-GTCA, 29200 Brest, France; 2 Physical Chemistry I & Research Center of Micro- and Nanochemistry and (Bio)Technology (Cμ), Department of Chemistry and Biology, University of Siegen, 57076 Siegen, Germany; 3 Unité Mixte de Recherche (UMR), Centre National de la Recherche Scientifique (CNRS) 6521, Université de Brest (UBO), CS 93837, 29238 Brest, France; 4 Macromolecular Chemistry, Department of Chemistry and Biology, University of Siegen, 57076 Siegen, Germany; 5 Coordination Chemistry Team, Unité Mixte de Recherche (UMR), Centre National de la Recherche Scientifique (CNRS) 7312, Institut de Chimie Moléculaire de Reims (ICMR), Université de Reims Champagne-Ardenne, BP 1039, CEDEX 2, 51687 Reims, France; 6 CHRU de Brest, Service de Génétique Médicale et de Biologie de la Reproduction, Centre de Référence des Maladies Rares Maladies Neuromusculaires, 29200 Brest, France

**Keywords:** antimicrobial photodynamic therapy, antimicrobial resistance, biofilm, benchmark analysis, cystic fibrosis, micro-environment, ruthenium complexes

## Abstract

Antimicrobial photodynamic therapy (aPDT) depends on a variety of parameters notably related to the photosensitizers used, the pathogens to target and the environment to operate. In a previous study using a series of Ruthenium(II) polypyridyl ([Ru(II)]) complexes, we reported the importance of the chemical structure on both their photo-physical/physico-chemical properties and their efficacy for aPDT. By employing standard in vitro conditions, effective [Ru(II)]-mediated aPDT was demonstrated against planktonic cultures of *Pseudomonas aeruginosa* and *Staphylococcus aureus* strains notably isolated from the airways of Cystic Fibrosis (CF) patients. CF lung disease is characterized with many pathophysiological disorders that can compromise the effectiveness of antimicrobials. Taking this into account, the present study is an extension of our previous work, with the aim of further investigating [Ru(II)]-mediated aPDT under in vitro experimental settings approaching the conditions of infected airways in CF patients. Thus, we herein studied the isolated influence of a series of parameters (including increased osmotic strength, acidic pH, lower oxygen availability, artificial sputum medium and biofilm formation) on the properties of two selected [Ru(II)] complexes. Furthermore, these compounds were used to evaluate the possibility to photoinactivate *P. aeruginosa* while preserving an underlying epithelium of human bronchial epithelial cells. Altogether, our results provide substantial evidence for the relevance of [Ru(II)]-based aPDT in CF lung airways. Besides optimized nano-complexes, this study also highlights the various needs for translating such a challenging perspective into clinical practice.

## 1. Introduction

Photodynamic therapy (PDT) consists of administrating a non-toxic photosensitive molecule, called photosensitizer (PS), followed by light irradiation to generate reactive oxygen species (ROS) including singlet oxygen molecules (^1^O_2_) producing multiple non-specific alterations to living organisms in an area to treat [1,2,3]. This three-partners (O_2_, light and PS) reaction may be used in a wide range of biomedical settings, notably for anticancer and antimicrobial applications [2,4,5]. Antimicrobial PDT (aPDT) has been suggested as a promising alternative or complementary treatment to current antibiotic therapy [3,6,7,8,9]. Indeed, since aPDT does not require any interaction with a specific molecular target and may be more resilient than the latter, it could allow overcoming the increasing spread of multidrug-resistant (MDR) micro-organisms [6,7,8,10]. Although many in vitro investigations have been done ascertaining the potential relevance of such a strategy, much fewer studies have been conducted so far under in vivo infected conditions [11,12]. However, the activity of PS strongly depends on the environment (where the therapeutic effect must occur), which should be thus considered as an additional critical parameter in PDT [13].

Metal complexes constitute a vast pipeline of potential active drugs with great promise for various biomedical applications [14,15,16]. As recent examples, silver *N*-heterocyclic carbenes with long *N*-alkyl chains [17], titanium(IV) complexes [18] or platinum cyclo-octadiene complexes [19] have been reported for their antibacterial properties. Regarding ruthenium complexes, ruthenium(II) polypyridyl complexes (hereafter abbreviated as [Ru(II)]) are endowed with peculiar properties, making them promising chemotherapeutic candidates [20,21,22]. They constitute attractive PS for PDT, notably because (i) they strongly absorb visible light, (ii) they display tunable photophysical properties, (iii) they can efficiently produce ROS upon light irradiation and (iv) they are generally not cytotoxic in the dark [23,24,25]. Recently, we conducted a structure–activity study using a variety of [Ru(II)] and a series of Gram-positive or Gram-negative clinical bacterial strains [23]. The molecular structure of [Ru(II)] was shown to strongly impact their photophysical properties and in turn their ability to perform bacterial photo-inactivation. Notably, the polar and amphiphilic characters of asymmetric complexes were associated with both a higher affinity for bacteria and a greater aPDT efficiency. This in vitro study emphasized the phototoxic potential of [Ru(II)] against various bacteria, irrespective of their antibiotic resistance profile, and thus their relevance to overcome antimicrobial resistance (AMR). Of note, the clinical bacterial strains included in that study were responsible for diverse infections in humans, notably in the airways of cystic fibrosis (CF) patients.

CF (OMIM 219700) is a lethal inherited disorder frequent in Caucasian populations [26]. It is generally due to mutations occurring in the CF transmembrane conductance regulator (CFTR) gene, which encodes a protein channel inserted in the apical membrane of epithelial cells, where it regulates the luminal secretion of chloride and water. In CF patients, the pathophysiological disorders occurring in lung airways and the resulting pulmonary symptoms strongly impact their lifespan and quality of life [27]. The dysregulation of CFTR and other ionic channels leads to the dehydration of the airway surface liquid (ASL), with the consequent impairment of mucociliary clearance and the accumulation of a hyperviscous mucus [28]. This provides appropriate conditions for lung colonization by opportunistic pathogens, especially *Staphylococcus aureus* and *Pseudomonas aeruginosa* growing in the form of hard-to-treat biofilms [27].

As a first line to treat CF lung infections, antibiotherapy has significantly increased the quality of life and the median survival age of patients. Nevertheless, pulmonary physiological impairments (especially sticky sputum formation, lowered pH and osmotic and oxidative stresses) alter the efficiency of antibiotics by reducing their stability and diffusion, leading to sub-inhibitory concentrations in sputum [29,30]. Furthermore, bacteria in biofilms can resist to 100–1000 times higher doses of antibiotics compared with the same bacteria growing as planktonic cells [31]. In contrast with systemic antibiotic administration, aerosolized and nebulized deliveries allow for significant improvements (up to 100 times) of their concentrations in CF sputum [32,33,34]. However, continuous antibiotherapies increase the risk of resistance emergence that can severely limit the remaining antibiotic options in patients with advanced diseases [35]. Today, MDR micro-organisms, notably belonging to the WHO priority pathogen list [36], are increasingly reported in CF patients [37,38]. For all these reasons, CF lung disease can be considered a challenging model system to develop novel antimicrobial treatment targeting infected airways. To our knowledge, besides many other antimicrobial approaches, the potential of aPDT has been scarcely considered in that respect to date.

**Figure 1:**
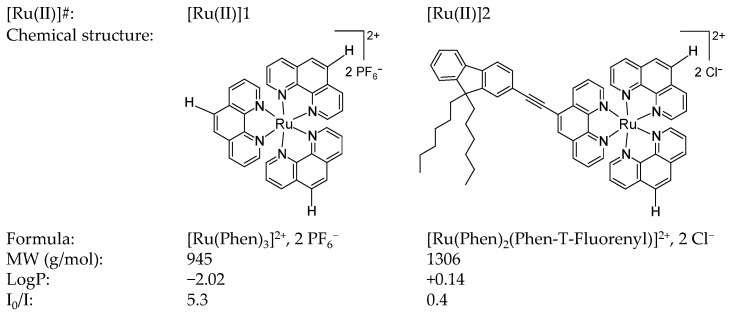
Chemical structure and relevant features of [Ru(II)] used in this study. The partitioning index LogP is a measure of the difference in solubility of the compound in 1-butanol and in water. I_0_/I is a measure of the singlet oxygen production efficiency, as determined using a Stern–Volmer analysis. LogP and I_0_/I were calculated and reported in our previous study (in which the counter-ion was hexafluorophosphate for both compounds) [23]. “Phen”: 1,10-phenanthroline.

The present study aimed to further expand our previous study on [Ru(II)] complexes [23], prospecting for the use of such compounds for performing aPDT in CF lung airways. Thus, two compounds (hereafter noted as [Ru(II)]1 and [Ru(II)]2, respectively; Figure 1) were selected considering their photo-physical and physico-chemical properties. They were used to conduct evaluations while taking into account CF pathophysiological disorders that may impact their activity (Table S1). In light of the results obtained, the relevance of [Ru(II)]-based aPDT for treating CF lung infections is critically discussed.

## 2. Materials and Methods

### 2.1. Materials

#### 2.1.1. Photosensitizers

Two [Ru(II)] were used as PS, i.e., [Ru(Phen)_3_]^2+^ (PF_6_^−^)_2_ and [Ru(Phen)_2_(Phen-T-Fluorenyl)]^2+^ (Cl^−^)_2_, hereafter abbreviated as [Ru(II)]1 and [Ru(II)]2, respectively (Figure 1). [Ru(II)]1 was purchased from StremChemicals (CAS. 60804-75-3, StremChemicals, Newburyport, MA, USA), whereas [Ru(II)]2 was synthetized as previously reported [23]. For both compounds, powders and solutions were stored in the dark at room temperature (RT). Unless otherwise stated, stock solutions were prepared in DMSO at 5 mM. In every test, [Ru(II)] were tested for concentrations ≤ 64 µM, thus bringing DMSO to final concentrations that did not exceed 1%, as recommended in CLSI guidelines.

#### 2.1.2. Illumination Set-Up and Light Treatment Procedure

**Figure 2:**
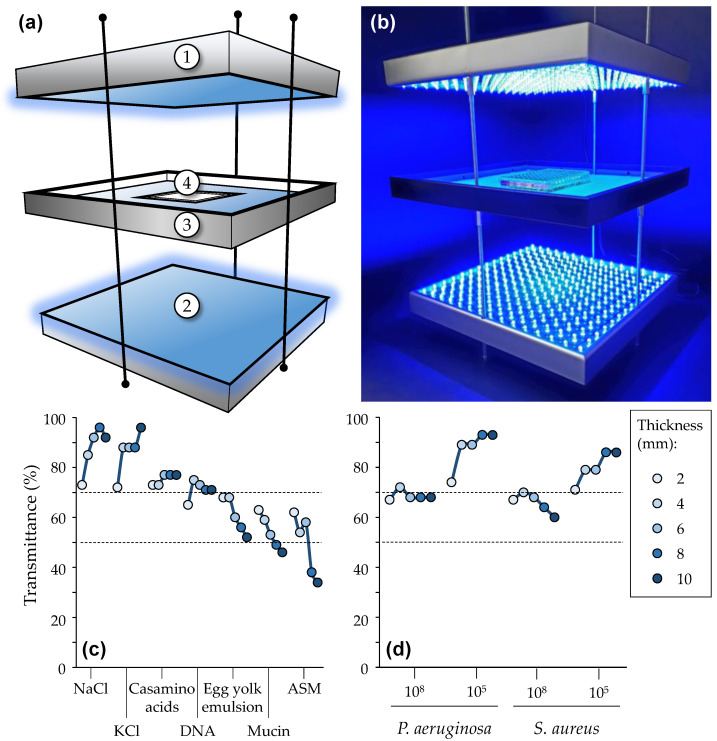
Illumination set-up. Schematic representation (**a**) showing the two LED panels (① and ②) placed above and underneath an in-between plate (③) used to hold materials placed in a central exposure area (④). The picture in (**b**) shows the illumination of a 96-well plate containing [Ru(II)] samples. Fluorescence was clearly visible to the naked eyes. Light transmittances was measured through various samples with increasing thicknesses, including ASM and its individual components (**c**) as well as several bacterial inoculums prepared in saline (**d**). The dashed lines correspond to 50 and 70% transmittances, respectively. See Figure S1 for more technical details about this setup.

Illumination treatments were performed using a home-made illumination device (Figure 2). This custom-built experimental system was designed to perform simultaneous top and bottom illumination with two wide (310 × 310 mm) panels (each incorporating 225 14W pure blue LEDs; λ: 450–470 nm) facing each other at adjustable distances. LED panels were placed on both sides of a plate that could be used to expose standard multi-well plates in a central exposure area (CEA). The heating resulting from light emission was measured using a temperature sensor placed in the middle of the CEA and monitoring temperature variation during irradiation. A PM160 wireless handheld power meter (Thorlabs, Bergkirchen, Germany) was used to measure light transmittance in various experimental settings, as detailed in Results section.

Unless otherwise stated, the illumination device was used for light treatment, as follows. A total of 20 min after the addition of [Ru(II)], the samples to irradiate were placed in the CEA, and then top and bottom exposure to LED light was repeated twice for 5 min, with 5 min under ambient light in between each exposure. The whole procedure was carried out at room temperature. A typical irradiation cycle thus lasted no more than 15 min in total (which was found to be sufficient to obtain PDT effects in many cases). The effect of that treatment was assessed by comparing samples subjected to light with the same samples kept in the dark. In this manuscript, the above-mentioned conditions are denoted “ON” (light-treated condition) and “OFF” (non-irradiated control), respectively.

#### 2.1.3. Bacterial Strains

A series of *S. aureus* or *P. aeruginosa* strains were used in this study: the laboratory strain *S. aureus* RN4220 [39], the methicillin-resistant *S. aureus* (MRSA) N315 [40], the *P. aeruginosa* (Schroeter) Migula (ATCC^®^ 19660™, hereafter noted PA19660) and the CF-originating *P. aeruginosa* PAH [41]. All bacterial routine handlings were conducted with Luria Bertani (LB) broth at 37 °C. Detailed information about each strain is available in the Supplementary Materials (Table S2).

#### 2.1.4. Cells

A new bioluminescent cell line was derived from the human CF bronchial epithelial cell line CFBE41o- [42]. The protocol used to establish this genetically modified cell line (hereafter noted CFBE-Luc) is detailed in the Supplementary Materials. They were grown in Eagle’s Minimum Essential Medium (EMEM) supplemented with 10% fetal bovine serum, 1% antibiotic and 1% L-glutamine (complete culture medium hereafter noted EMEMc; all components obtained from Lonza, Levallois-Perret, France). Incubations were performed at 37 °C in a humidified atmosphere containing 5% CO_2_.

#### 2.1.5. Specific Media

Artificial sputum medium (ASM) was prepared as previously reported [43]. Mannitol Salt Agar (Thermo Fischer Scientific, Hampshire, UK) and Pseudomonas Isolation Agar (Sigma Aldrich, St. Louis, MO, USA) were used to specifically determine the number of colony forming units (CFUs) of *S. aureus* and *P. aeruginosa*, respectively. Acetate buffers at various pH were prepared as described in the Supplementary Materials (Table S3). Saline solution (hereafter noted 1X NaCl) corresponded to 0.9% NaCl (Versol, London, UK).

### 2.2. Bacteria and Cell Culture Conditions

#### 2.2.1. Bacteria Growing as Planktonic Cells

Overnight cultures of the bacteria were prepared by inoculating 5 mL of LB broth with a single colony picked from an LB agar plate, followed by incubation at 37 °C for 12–16 h. For experiments requiring bacteria in the exponential phase, 10 µL of overnight culture were used to inoculate 10 mL of LB broth, with subsequent incubation for approximately 5 h at 37 °C. After centrifugation at 2000× *g* for 10 min at 4 °C, the supernatant was carefully discarded to isolate bacterial pellets. The latter were suspended in required solutions at the desired bacterial density, assuming that OD600 of 0.6 and 1.2 corresponded to 10^7–8^ CFU/mL for *P. aeruginosa* and *S. aureus*, respectively [23].

#### 2.2.2. Bacteria Growing as Sessile Cells in Biofilms

*S. aureus* and *P. aeruginosa* overnight cultures were prepared as mentioned in the previous section. After the centrifugation and resuspension of bacterial pellets in LB, 10 mL of diluted suspension was prepared (at 10^6^ CFU/mL) and then used to inoculate a 96-well round bottom microplate (Costar, Kennebunk, MI, USA). Incubation was then carried out for 20 h at 37 °C under static humidified conditions.

#### 2.2.3. Co-Cultures of Bacteria and Eukaryotic Cells

Co-culture experiments involving eukaryotic and prokaryotic cells were conducted using CFBE-Luc and PA19660, respectively. Briefly, CFBE-Luc were first grown (without bacteria) under either of the two following types of culture conditions. (i) For cultures in submerged conditions, cells were seeded in 96-well plates (Sarstedt, Numbrecht, Germany) at a density of 50,000 cells in 200 µL of EMEMc per well and were incubated for 24 h at 37 °C before subsequent use. (ii) For cultures at the air–liquid interface (ALI), cells were seeded in 0.4 µm pore membrane inserts (Sarstedt, Numbrecht, Germany) at a density of 150,000 cells in 200 µL of EMEMc per insert and were incubated at 37 °C. After 1 week, the culture medium in the apical chamber was removed, and the cells were maintained at ALI for at least 3 weeks, with the culture medium in the basal chamber being renewed every 3–4 days. Trans-epithelial electrical resistance was measured using an EVOM2 epithelial Volt/Ohm meter (World Precision Instruments, Hertfordshire, Germany).

### 2.3. Characterizations of [Ru(II)] under Various Experimental Conditions

All below-mentioned fluorescence and absorption measurements were performed using the multiplate reader Mithras2 LB 943 (Berthold Technology, Thoiry, France).

#### 2.3.1. Fluorescence and UV Visible Spectra

For fluorescence measurements, 50 µM [Ru(II)] in 200 µL was introduced per well of a black 96-well plate (Thermo Fischer Scientific, Roskilde, Denmark). Measurements were performed with excitation at 460 ± 15 nm and emission readings at 590 ± 22 nm. For UV-visible spectra, UV/vis transparent flat-bottom multi-well plates (Greiner Bio-One, Frickenhaussen, Germany) were used with 50 µM [Ru(II)], in either 1X NaCl or water. Absorption was measured from 230 to 630 nm (increment = 5 nm) at 25 °C.

#### 2.3.2. PS interaction with Bacteria

Bacteria (10^8^ CFU/mL) were mixed with 50 µM [Ru(II)] in water or saline. A first measurement was performed to determine the total fluorescence. Then, a centrifugation at 2000× *g* for 10 min at 4 °C was performed to pellet bacterial cells. The supernatant and pellet were carefully isolated, and a second fluorescence measurement was performed on these two fractions. The fluorescence distribution in the latter informed about the strength of the interaction between [Ru(II)] and the bacteria (assuming that the higher fluorescence in the pellet, the stronger interaction of [Ru(II)] with bacteria).

#### 2.3.3. PI Assay

Bacteria (10^8^ CFU/mL) were mixed with 50 µM [Ru(II)] in saline. Following the centrifugation of samples at 2000× *g* for 10 min at 4 °C, the bacterial pellets were isolated and suspended in 1 mL of 100 µM propidium iodide (PI; Sigma Aldrich, St. Louis, MO, USA). As a positive control of permeabilization, bacteria previously treated with 70% isopropanol were subjected to the same. These mixtures were incubated for 5 min at RT. Following another centrifugation, the supernatant was discarded, and bacterial pellets were suspended in 1X NaCl. Fluorescence was read with excitation at 488 ± 8 nm and emission at 617 ± 15 nm.

#### 2.3.4. Determination of Singlet Oxygen Production

The singlet oxygen sensor green (SOSG) fluorescent probe (Fisher Scientific, Eugene, OR, USA) was used to quantify ^1^O_2_. Briefly, 50 µL of 50 µM [Ru(II)] was mixed with 50 µL of Tris buffer at pH 7.0, supplemented or not with 10^7^ CFU/mL of bacteria. Then, 10 µL of SOSG was added to reach a final concentration of 10 µM. After incubation for 20 min in the dark, light treatment was performed before reading fluorescence, with excitation at 504 ± 6 nm and emission at 525 ± 12 nm.

#### 2.3.5. Determination of Intracellular ROS Production

The 2′,7′-dichlorofluorescin diacetate (DCFH-DA; Merck, Darmstadt, Germany), a cell permeant reagent fluorogenic dye, was used to quantify intracellular ROS production. It is converted in DCFH carboxylate anion (DCFH), a non-fluorescent compound, by cellular esterase. In the presence of ROS (e.g., hydroxyl anion or hydrogen peroxide), it is oxidized in dichlorofluorescein (DCF), which is highly fluorescent. Briefly, 10^8^ CFU/mL of bacteria were exposed to 10 µM of DCFH-DA in 1X PBS for 30 min. Bacteria were then collected by centrifugation at 2000× *g* for 10 min at 4 °C and were washed with 1X NaCl. Another centrifugation was performed, and bacterial pellets were mixed with 200 µL of 25 µM [Ru(II)] in 1X NaCl. After irradiation, fluorescence was measured with excitation at 485 ± 12 nm and emission at 535 ± 12 nm.

#### 2.3.6. Hypoxic Condition Assay

Sunflower oil (Merck, Darmstadt, Germany) was deposited at the surface of a given medium to prevent any contact with ambient air. Janus Green B (JGB, Merck, Darmstadt, Germany) mixed in the medium (0.03 %, *w*/*v*) was used as a fluorescent hypoxic indicator, as previously reported [44]. More details are given in the Supplementary Materials (Figure S7).

### 2.4. Assessment of Antibacterial PDT Effects

#### 2.4.1. Assessment of PDT Effects on Planktonic Cells

The determination of the bacteria still alive following a given treatment was performed using several methods. First, the spotting method provided a rough demonstration of the specific effect of light and any possible dark toxicity. For doing so, 5 µL of each condition (just before and then after light treatment) was dropped on a nutritive (non-selective) agar plate using a multichannel pipette. Following the incubation of the plates overnight at 37 °C, it was possible to distinguish between full, intermediate or no antibacterial effect compared to control conditions. Second, semi-quantitative bacterial counting was performed to roughly estimate the number of CFU/mL. For doing so, control and test conditions were subjected to serial 10-fold dilutions (10 µL in 90 µL of 1X NaCl), and then 5 µL of each dilution was spotted on an LB agar plate. After incubation at 37 °C for 24–48 h, counting was performed to estimate the bacterial density in each sample. Third, growth kinetics allowed for the estimation of reductions in bacterial density down to 10^5^ CFU/mL, as previously reported [23]. Briefly, 195 µL of LB liquid medium per well was inoculated with either 5 µL of a given test condition or 5 µL of serial dilutions of a control inoculum of the same bacteria. The plate was then introduced into the Mithras2 LB943 microplate reader (Berthold). Incubation was carried out at 30 °C for at least 16 h, with stirring and OD readings (at 600 nm) being repeated every 10 min. The number of viable bacteria was inferred by comparing the kinetics of test conditions with those of the control inoculums (containing from 10^5^ to 10^0^ CFU/mL) [23].

#### 2.4.2. Assessment of PDT Antibiofilm Effects

PDT eradication assays were conducted following protocols reported earlier [45], with some modifications. We distinguished two experimental set-ups, which we named pre-delivery and post-delivery, depending on the timing of the addition of [Ru(II)] to bacteria with respect to the biofilm formation. Briefly, for the pre-delivery eradication assay, *S. aureus* RN4220 or *P. aeruginosa* PA19660 at a density of 10^6^ CFU/mL was first mixed with a given [Ru(II)] at 50 µM in 0.5X NaCl. After incubation for 20 h at 37 °C under static conditions, the planktonic phase was carefully discarded, and the isolated biofilm was subjected to light treatment. The latter was performed using a modified procedure, with 15 min instead of 5 min at each step (to allow for obtaining better PDT effects). For the post-delivery eradication assay, the above-mentioned bacteria were grown (without [Ru(II)]) for 20 h at 37 °C under static conditions. Then, the planktonic phase was discarded, and the biofilm was carefully washed twice with saline solution before being covered with 100 µL of 50 µM [Ru(II)] in 0.5X NaCl. After incubation for 20 min, light treatment was performed as detailed above. For both eradication assays, antibacterial effects were assessed immediately after light treatment using the procedures described above (see Section 2.4.1) and in the Supplementary Materials. Ciprofloxacin (Sigma Aldrich, St. Louis, MO, USA) at a dose of 16 µg/mL was used as a positive control.

#### 2.4.3. Assessment of PDT Effects on Bacteria in Co-Culture Experiments

Following the cell culture in either of the two above-mentioned culture conditions (see Section 2.2.3), 50 µL of bacteria (10^5^ CFU/mL of PA19660) mixed with either of the two [Ru(II)] compounds (at 50 µM in 0.5X NaCl) were deposited over CFBE-Luc cells. The light treatment was performed 15 min later. Then, the mixture containing bacteria and [Ru(II)] was removed, and the bacterial load was immediately determined by enumeration. The culture medium of submerged cells or cells cultured at ALI was replaced with fresh antibiotic-free medium. After 24 h incubation, the cells were lysed with the Passive Lysis Buffer (Promega, Charbonnières-les-Bains, France). For every test, luciferase expression and total protein content were determined using the Luciferase Assay System (Promega, Charbonnières-les-Bains, France) and the BC assay kit (Uptima, Interchim, Montluçon, France), respectively. The data were expressed as relative light units (RLU) per milligram of total proteins, as previously reported [41].

### 2.5. Statistical Analysis

For every assay, tests were performed in technical triplicates (*n* = 3), and at least 2 independent experiments were performed (N ≥ 2). The results presented correspond to the mean and standard deviation (mean ± SD) for all the variables. Data were compared by using Student’s *t*-test processed with Prism software version 6.00 (GraphPad, San Diego, CA, USA). Statistically significant differences were denoted as follows: ***, *p*-value ≤ 0.001; **, *p*-value ≤ 0.01; *, *p*-value ≤ 0.05. Non-significant differences were noted “ns”.

## 3. Results

In this study, two [Ru(II)] (Figure 1) were compared in a series of assays, in an attempt to unveil the isolated influence of various factors showing relevance to the conditions in CF lung airways (Table S1). We first performed photophysical characterizations to assess both the illumination protocol and the [Ru(II)] selected. Then, various experimental conditions were considered to underpin and weigh the parameters that could interfere with [Ru(II)]-mediated aPDT.

### 3.1. Characterizations of the Illumination System and PS

#### 3.1.1. Assessment of the Functioning of the Illumination System

All light treatments in this study were performed using a custom-built illumination system made of light-emitting diodes (LED) specifically suited for the [Ru(II)] evaluated (Figure 2A). LED panels were set equidistant (15 cm) from an in-between plate bearing a central exposure area (CEA). This allowed us to obtain an effective homogenous illumination field over wide areas, with a maximum of ~ 4 mW/cm² (or mJ/s/cm²) and less than 10% variation of the light power measured in different locations of the CEA (Figure S1). The heating induced by the illumination was monitored, showing that the temperature increase as a function of illumination time was moderate and negligible in every case (Figure S1). Indeed, the light treatments performed in this study (consisting of 5 to no more than 15 min of continuous irradiation) led to an estimated temperature increase lower than 2 °C. Even after 1 h of continuous illumination, an elevation of no more than 4 °C above RT could be measured. Importantly, results were the same whether the temperature probe was placed in the CEA in the air or in a liquid (not shown). Thus, it can be stated that the short-lived irradiations performed herein did not induce any noticeable temperature increase in the samples, and light-induced heating did not play any significant role in the results obtained.

Furthermore, light transmittance was assessed through liquid samples of various thicknesses, turbidities and compositions (Figure 2B). For practical reasons, the light emitted from one panel (either the top or the bottom one) was measured after its diffusion through liquid samples contained in a wider well than the diameter of the surface active captor of the power meter (i.e., 1.55 cm and 0.95 cm, respectively). In particular, we studied NaCl, KCl, casamino acids, egg yolk emulsion and mucin, as well as their combination in ASM [43]. It was found that light transmittance was the most affected by ASM, mainly due to its content in mucin and egg yolk emulsion, causing increased light scattering. Then, the inoculum of bacterial samples was also shown to curb light diffusion, regardless of the bacteria considered. Overall, light transmittance was lowered when the medium was more turbid, complex or viscous. Other measurements demonstrated the benefit of top and bottom illumination to better irradiate samples in their entirety (not shown). All combined, these trials qualified our system for a controlled screening under multiple experimental conditions.

#### 3.1.2. Photophysical Characterizations of [Ru(II)]

**Figure 3:**
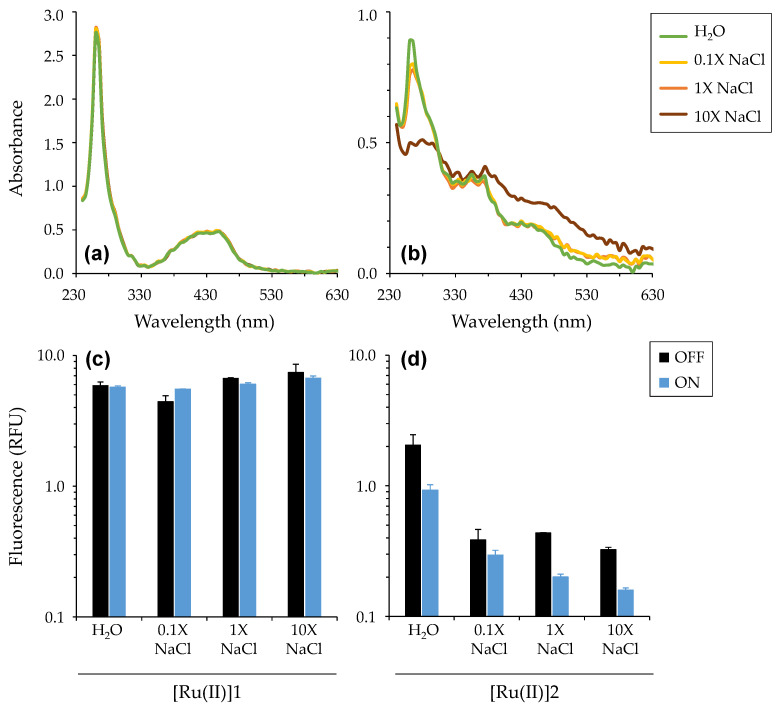
Photophysical properties of [Ru(II)] in water or in saline solutions. Absorption in the UV-visible spectrum (**a**,**b**) and fluorescence (**c**,**d**) were determined with [Ru(II)]1 (**a**,**c**) and [Ru(II)]2 (**b**,**d**). In every test, [Ru(II)] concentration was 50 µM in 200 µL. Fluorescence measurements were performed before (OFF) then after (ON) light treatment. Mean ± SD with *n* = 3. The fluorescence background is ~0.05 RFU in every case. “RFU”: relative fluorescence unit.

UV-visible absorption and fluorescence measurements were determined for both [Ru(II)], either in water or in saline solutions of different ionic strengths, before then after light treatment (Figure 3 and Figure S2).

Considering UV-visible absorption, the two compounds exhibited clearly different spectra, which did not vary in the same direction when increasing the salt concentration in the solution. (1) In water, [Ru(II)]1 showed (i) an intense band between 250 and 280 nm, due to an intra-ligand (IL) electronic transition, (ii) a band around 300–400 nm likely attributed to an intra-ligand charge transfer (ILCT) involving the phenanthroline unit and (iii) a broad band around 450 nm corresponding to metal-to-ligand charge transfer (MLCT) characteristic of such polypyridyl ruthenium complexes. The large width of the MLCT band is attributable to vibronic broadenings and/or the overlap of more bands corresponding to different close-lying electronic transitions [46]. Compared with [Ru(II)]1, [Ru(II)]2 exhibited a strongly different UV-visible spectrum, characterized with lower absorption at every wavelength, but exhibiting clearly visible bands. (2) In saline solutions, [Ru(II)]1 spectra were almost indistinguishable, even for high salt concentrations (up to 10X NaCl). In contrast, [Ru(II)]2 showed more sensitivity to saline, which could be detected right from the lowest concentration tested (i.e., 0.1X NaCl). For 10X NaCl, the spectrum was smoothed with less visible bands. Overall, spectra for both compounds as measured in water or saline (up to 0.9% i.e., 1X NaCl) were very similar to those reported earlier in 1X PBS [23]. Noticeably, they were unchanged following light treatment (Figure S2).

Considering fluorescence, [Ru(II)]1 was found to be more fluorescent than [Ru(II)]2, irrespective of the condition considered. Furthermore, whereas [Ru(II)]1 fluorescence intensity was almost the same in every condition, it was reduced by about half for [Ru(II)]2 in saline conditions. Light treatment also provided distinct results depending on the compounds, with [Ru(II)]1 fluorescence remaining the same whereas it was significantly reduced for [Ru(II)]2. These results may be ascribed, at least in part, to the difference in solubility of these two complexes, as denoted by their respective LogP value (Figure 1), resulting in obvious (clearly visible) turbidity, light scattering and lesser light diffusion occurring for [Ru(II)]2 in saline solutions. The lowered fluorescence signal specifically noted for [Ru(II)]2 after light treatment, in water or saline, is also remarkable, but not explained at present. Importantly, all measurements were performed over a period that did not permit any noticeable precipitation.

All combined, these results further point out that the molecular engineering of such complexes can have strong impacts on their physico-chemical and photophysical properties. This is especially verified under saline conditions approaching those in CF lungs (Table S1).

#### 3.1.3. Singlet Oxygen and ROS Productions

**Figure 4:**
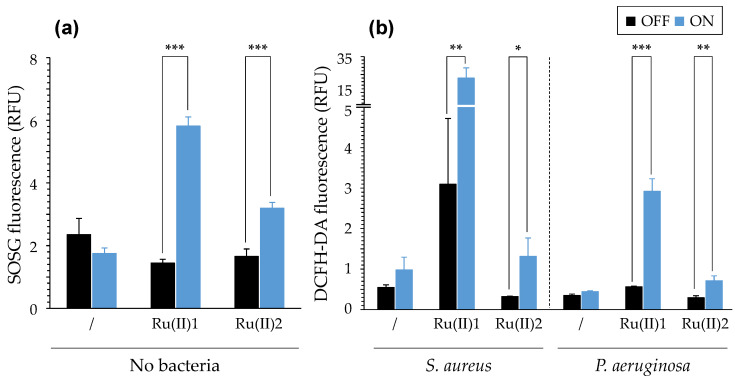
Production of singlet oxygen and intracellular ROS upon light treatment of [Ru(II)]. Results were obtained using SOSG (**a**) and DCFH-DA (**b**), respectively; *S. aureus* and *P. aeruginosa* were RN4220 and PA19660, respectively. [Ru(II)] = 25 µM in Tris-HCl for SOSG and 1X NaCl for DCFH-DA. Mean ± SD with *n* = 3 (representative results of N = 2). ***, *p*-value ≤ 0.001; **, *p*-value ≤ 0.01; *, *p*-value ≤ 0.05. See Figure S3 for more data.

The abilities of [Ru(II)] to produce ^1^O_2_ and ROS were investigated in various experimental conditions. For ^1^O_2_ production, we used the cell impermeant derivative SOSG probe, which is highly selective for ^1^O_2_ and does not show any appreciable response to ^●^OH or ^●^O_2_^−^, as claimed by the manufacturer. The SOSG functioning primarily relies on O_2_ availability and can be used in the absence or presence of cells. Upon light treatment, in the absence of bacteria, the production of ^1^O_2_ was detectable with [Ru(II)]1 and to a lesser extent with [Ru(II)]2 (Figure 4). In the presence of bacteria, this production was also detected, and it was found to be enhanced for [Ru(II)]2 with *S. aureus* (Figure S3). Regarding ROS production, we used the DCFH-DA probe that is cell-permeable and requires the use of live cells. Upon entering a bacterial cell, DCFH-DA is hydrolyzed by esterases to the non-fluorescent 2′,7′-dichlorodihydrofluorescein, which can be then oxidized by a variety of ROS to the strongly fluorescent dichlorofluorescein. Despite some background and signal variations, it was noticeable that both compounds were able to induce ROS production, as demonstrated by the fluorescence increase measured after light treatment. [Ru(II)]1 appeared to be more efficient than [Ru(II)]2 for both ^1^O_2_ and ROS productions, which could be stronger in the presence of *S. aureus* than *P. aeruginosa*. These findings were actually in accordance with findings we reported earlier using other experimental determination methods (Figure 1) [23].

#### 3.1.4. Bacteria/Ru(II) Interaction and PI Assay

**Figure 5:**
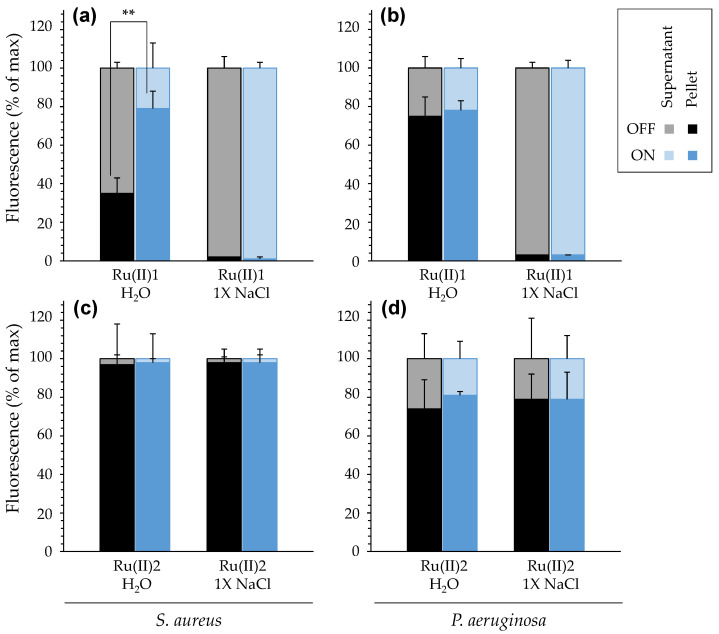
Interaction assay between [Ru(II)] and bacteria when mixed either in water or in 1X NaCl. Results were obtained using [Ru(II)]1 (**a**,**b**) or [Ru(II)]2 (**c**,**d**) with *S. aureus* RN4220 (**a**,**c**) and *P. aeruginosa* PA19660 (**b**,**d**). See Figure S4 for results obtained with other bacterial strains. [Ru(II)] = 25 µM. Mean ± SD with *n* = 3 and N ≥ 2. **, *p*-value ≤ 0.01.

The capacity of [Ru(II)] to interact with bacteria in solution was evaluated by considering the ability of the former to absorb at the surface of—and co-precipitate with—the latter, as reported earlier [23]. Fluorescence measurements were performed to track [Ru(II)] after a centrifugation step. In the absence of bacteria, the fluorescence in the solution did not vary, and no pellet was formed, meaning that [Ru(II)] did not precipitate in any condition considered. On the other hand, in the presence of bacteria, the centrifugation led to pellet the latter, and the fluorescence in the solution could decrease, reflecting an interaction between bacteria and [Ru(II)]. As shown in Figure 5, very different results were obtained with the two [Ru(II)] assayed, depending on the strains, the ionic strength and, in some cases, the light applied. For [Ru(II)]2, it was noteworthy that it fully precipitated with bacteria in almost every condition considered, showing its propensity to interact with *S. aureus* and (to a slightly lesser extent) with *P. aeruginosa*. When considering [Ru(II)]1, the salinity prevented its capacity to interact with any bacteria, whereas in water, some interaction could be measured, with various intensities depending on the bacteria considered. Noticeably, light treatment (performed before the centrifugation step) was found to significantly increase the interaction capacity of [Ru(II)]1 when mixed in water with either of the two *S. aureus* strains evaluated (but not with *P. aeruginosa* strains; Figure 5 and Figure S4).

We also investigated several possible effects of [Ru(II)] on the integrity of bacterial cells. First, the surface charge of bacteria did not change following the mixture with a given [Ru(II)] in water, neither before nor after light treatment (Figure S5). Indeed, in every case, *S. aureus* and *P. aeruginosa* exhibited zeta potentials of around −15 mV and −20 mV, respectively. Second, the ability of [Ru(II)] to permeabilize bacterial cells was evaluated by conducting a PI assay. Live cells are normally non-permeable to PI, which can thus be used as a dye to detect dead cells in a population. This highlighted again that the two [Ru(II)] yielded different results in the experimental conditions considered. Indeed, no obvious permeabilization could be found with [Ru(II)]1 when mixed with *S. aureus* or *P. aeruginosa*. On the other hand, whereas no effect was found towards *P. aeruginosa*, [Ru(II)]2 could permeabilize *S. aureus* cells, which was clearly observed when light treatment was applied (Figure S5).

### 3.2. Impact on aPDT of Different Parameters during Light Treatment

The possible impact on the photodynamic inactivation of various parameters relevant with respect to CF lung condition was evaluated in dedicated experiments. In every case, bacteria were mixed with either [Ru(II)] and subjected to light treatment in the specific conditions detailed below. Immediately after, the remaining viable cells were determined by growing these under standard, non-selective conditions, as detailed in the Material and Methods section. This allowed us to study the potential impact of each parameter specifically during the duration of light treatment.

#### 3.2.1. Impact of Salinity

The effect of salinity on the antibacterial effects mediated by either of the two [Ru(II)] was evaluated with two *S. aureus* and two *P. aeruginosa* strains. The results obtained showed that, in many cases, better aPDT effects (but also stronger light-independent toxicity) are obtained in water compared to saline solution (Table 1). With respect to the antibiotic-resistant profile of bacteria (Table S2), no correlation could be clearly shown with susceptibility to a given [Ru(II)], in accordance with previous studies [3,23]. These results further point out the critical importance of salinity on [Ru(II)]-mediated PDT, irrespective of the compound used.

Considering these results, subsequent experiments were conducted with adjusted saline and [Ru(II)] concentrations (as specified below), to allow for the highlighting of the impact of other parameters on aPDT.

#### 3.2.2. Impact of Acidic pH

**Figure 6:**
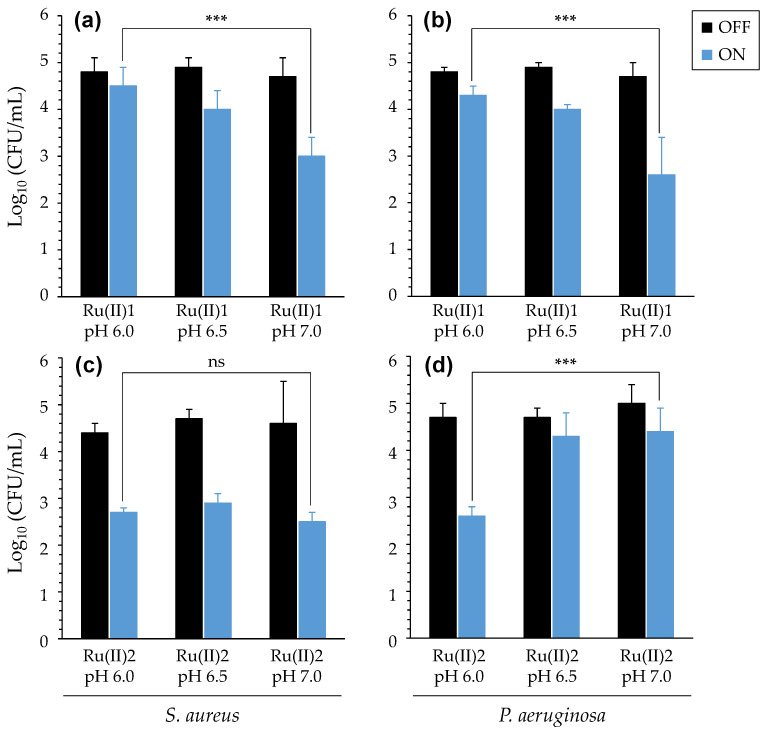
PDT effects of [Ru(II)] at various pH. Results were obtained using [Ru(II)]1 (**a**,**b**) or [Ru(II)]2 (**c**,**d**) with *S. aureus* RN4220 (**a**,**c**) and *P. aeruginosa* PA19660 (**b**,**d**). [Ru(II)] concentration was adapted to each strain evaluated: [Ru(II)]1 was 40 and 10 µM, whereas [Ru(II)]2 was 0.1 and 1 µM in acetate buffers, when assaying *S. aureus* and *P. aeruginosa*, respectively. Mean ± SD with *n* = 3 and N = 2. ***, *p*-value ≤ 0.001; “ns”: non-significant.

Bacteria were mixed with either [Ru(II)] in buffers at different pH (7.0, 6.5 or 6.0) encompassing the values commonly measured in the CF ASL (Table S1). After light treatment, bacterial density was determined to distinguish the possible effects of light and pH towards *P. aeruginosa* and *S. aureus*. First, it was checked that, for a given strain, the growth ability was not affected following transient incubation in the various conditions assayed (Figure S6). As shown in Figure 6, the pH could impact the antibacterial effects, depending on both the bacteria evaluated and the [Ru(II)] used. For [Ru(II)]1, significantly higher reductions in the density of bacteria (either *S. aureus* or *P. aeruginosa*) were measured at a neutral pH than at an acidic pH. For [Ru(II)]2, the PDT effects towards *S. aureus* were almost the same at the various pH investigated. Noticeably, better effects against *P. aeruginosa* could be obtained for the lowest pH, thus showing an opposite trend as compared with [Ru(II)]1.

These results thus underscore that the pH of the environment can indeed impact, in multiple ways, the PDT activity of the two [Ru(II)] considered herein.

#### 3.2.3. Impact of Reduced Oxygenation

Since CF patients suffer from impaired ventilation, and since oxygen is a pivotal parameter in PDT, the impact of reduced oxygenation was also important to study. First, we checked the possibility to achieve reduced oxygenation in solution by using oil deposition at the surface of the latter, with Janus Green B (JGB) being used as an oxygen indicator, as previously reported [44] (see Supplementary Materials Figure S7).

**Figure 7:**
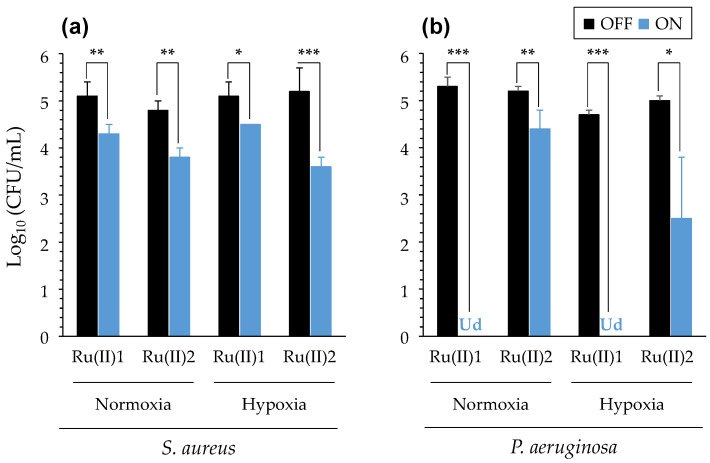
PDT effects of [Ru(II)] in normal or hypoxic media. Results were obtained with *S. aureus* RN4220 (**a**) and *P. aeruginosa* PA19660 (**b**). [Ru(II)]1 was 50 and 25 µM, whereas [Ru(II)]2 was 0.1 and 1.25 µM in 0.5X NaCl, when assaying *S. aureus* and *P. aeruginosa*, respectively. Mean ± SD with *n* = 3 (representative data of N = 4). ***, *p*-value ≤ 0.001; **, *p*-value ≤ 0.01; *, *p*-value ≤ 0.05. “Ud”: undetectable bacteria.

Then, the ability of both [Ru(II)] to achieve aPDT was compared in hypoxic versus normally oxygenated conditions (with or without oil deposition, respectively, with JGB being no longer used). As shown in Figure 7, it was remarkable that, in both conditions evaluated, [Ru(II)]1 and [Ru(II)]2 exhibited almost similar aPDT efficiencies towards *S. aureus* and *P. aeruginosa*. Although further investigations should be conducted, these results suggest that [Ru(II)] may exhibit PDT effects through an oxygen independent pathway, as suggested earlier for other PS by Hamblin and colleagues [47].

#### 3.2.4. Impact of Artificial Sputum Medium (ASM)

**Figure 8:**
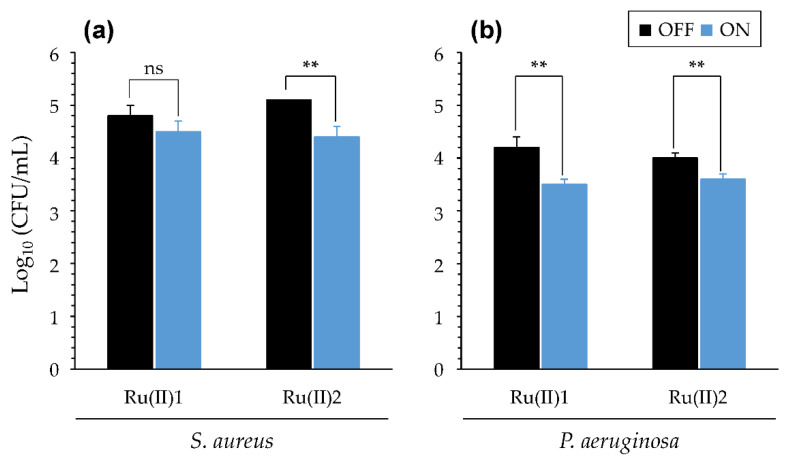
PDT effects of [Ru(II)] in ASM. Results were obtained with *S. aureus* RN4220 (**a**) and *P. aeruginosa* PA19660 (**b**) for prolonged irradiation. See Figure S8 for results obtained when using standard light treatment. [Ru(II)] concentration = 25 µM in 1X ASM. Mean ± SD with *n* = 3 and N = 2. **, *p*-value ≤ 0.01; “ns”: non-significant.

The ability of [Ru(II)] to exert aPDT effects on bacteria in ASM was also evaluated. As shown in (Figure S8), upon light treatment, no effects could be found with any [Ru(II)]. Taking into account the lesser ability of light to diffuse in ASM (Figure 2B), we adapted the procedure used for light treatment, using 15 min instead of 5 min at each step. Following such prolonged light treatment, some aPDT effects could be obtained (Figure 8). Indeed, in the experimental condition used, both [Ru(II)] slightly but significantly reduced the bacterial load of *P. aeruginosa*. On the other hand, only [Ru(II)]2 was able to do the same against *S. aureus*. Compared with the evaluations performed in other simpler media, ASM strongly impacted the ability of [Ru(II)] to exert aPDT. These results suggest that less efficient light penetration, but also some interaction of [Ru(II)] with some ASM component(s), could interfere with the ability of these PS to exert aPDT, reducing or abolishing the latter towards *P. aeruginosa* and *S. aureus*, respectively.

### 3.3. PDT towards Bacteria Grown in Different Conditions

#### 3.3.1. PDT towards Polymicrobial Cultures

The ability of [Ru(II)] to inhibit planktonic bacteria was evaluated, using standardized inoculums of *S. aureus* and *P. aeruginosa* strains. The latter were either used alone or were mixed at the same density, and then light treatment was performed either directly or after 4 h of co-cultivation. As shown in Figure S9, bacteria grew with the same efficiency in every condition considered, including in the presence of [Ru(II)], provided that light was not applied. However, when light treatment was performed, both [Ru(II)] were efficient to fully photo-inactivate *S. aureus*, irrespective of the culture condition, i.e., in mono and polymicrobial conditions. On the other hand, no aPDT effect was obtained towards *P. aeruginosa* in the experimental condition considered. However, the full eradication of both bacteria could be obtained when increasing [Ru(II)] concentrations (not shown).

#### 3.3.2. PDT towards Bacterial Biofilms

Antibiofilm PDT assays were performed considering the ability of [Ru(II)] to eradicate biofilms of either *S. aureus* or *P. aeruginosa*. For this purpose, the protocols originally described by Haney and colleagues [45] for assaying antibiotics or peptides were adapted for the evaluation of PS. In particular, a light treatment step was included, which here consisted of illuminating biofilm two times for 15 min, with an incubation time of 15 min under ambient light between each irradiation (“modified light treatment procedure”).

**Figure 9:**
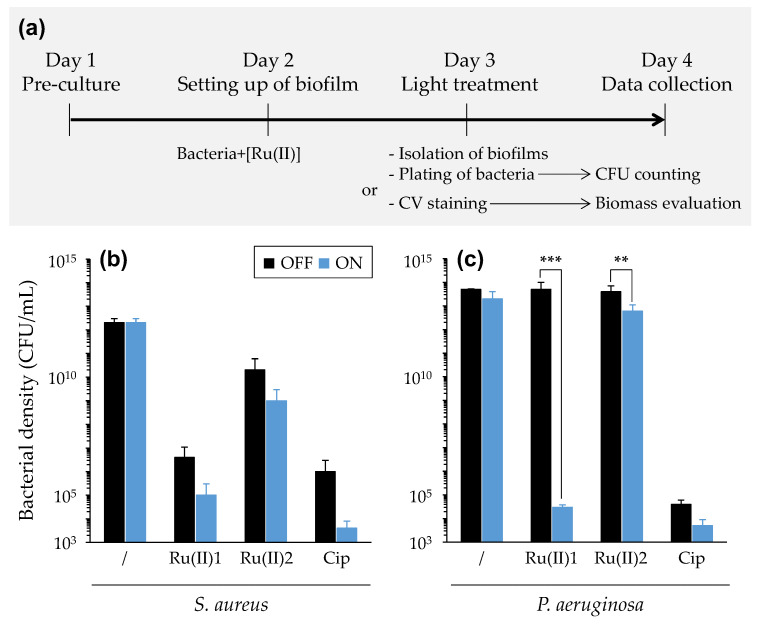
PDT effect of [Ru(II)] towards bacteria in biofilms (“pre-delivery antibiofilm assay”). The workflow of the experimentation is presented (**a**) with the results obtained when assaying *S. aureus* RN4220 (**b**) and *P. aeruginosa* PA19660 (**c**). Mean ± SD with *n* = 3 and N = 3. ***, *p*-value ≤ 0.001; **, *p*-value ≤ 0.01. “Cip”: ciprofloxacin.

A first assay was conducted to determine the potency of [Ru(II)] incorporated into biofilm, thus bypassing the presumably limiting diffusion step in the latter. For doing so, planktonic bacteria were first mixed with a given [Ru(II)] (but without doing any light treatment at this stage). After incubation for 20 h under static conditions, planktonic bacteria in the supernatant were carefully discarded, and then light treatment was applied. Following this, cell viability and biomass were determined by bacterial enumeration and crystal violet staining, respectively (Figure 9). For *S. aureus* biofilm, both [Ru(II)] demonstrated some toxicity in the absence of specific light treatment, with significant (*p* < 0.001) reductions in the bacterial load by six and two logs compared to biofilm control, respectively. The light treatment allowed enhancing this effect by one more log reduction. For *P. aeruginosa* biofilm, no dark toxicity was observed, with the same bacterial load being determined with or without any [Ru(II)] in the absence of light. Upon light treatment, the bacterial loads were reduced by nine and one log with [Ru(II)]1 and [Ru(II)]2, respectively (Figure 9). No difference with respect to biomass could be observed, regardless of the conditions evaluated (Figure S10).

In light of these encouraging results, complementary experiments were performed following a more conventional biofilm eradication assay [45,48]. Here, biofilm was settled using bacteria in the absence of any [Ru(II)]. Following incubation for 20 h under static conditions, the supernatant containing planktonic cells was discarded, and the biofilm was carefully washed before being exposed to either of the two [Ru(II)]. After light treatment was applied, antibiofilm effects were evaluated in different ways. First, the cell viability and metabolic state were determined with a resazurin assay (Figure S11). Upon light treatment, no significant difference could be noticed in “control” conditions (i.e., with biofilms without any [Ru(II)]), whereas a significant reduction in dye absorbance was found in “test” conditions (i.e., with biofilms treated with [Ru(II)]1), which was dose-dependent (i.e., stronger for 50 µM than 30 µM of compound) with both *S. aureus* and *P. aeruginosa*. Bacterial enumeration and CV staining were also carried out to further characterize biofilm eradication effects. Some reduction in the bacterial load in biofilm could be found, but with many variations (in difficult-to-reproduce experiments), and no modification of the total biomass could be underscored. Noticeably, the results suggest that the antibiofilm potency of [Ru(II)]1 following post-delivery may be superior to that of [Ru(II)]2, a finding in line with the results obtained in the “pre-delivery antibiofilm assay”.

#### 3.3.3. PDT towards Bacteria in the Presence of Eukaryotic Cells

Last, we investigated the ability of [Ru(II)] to display aPDT while not affecting a culture layer of eukaryotic cells. For this purpose, genetically modified human bronchial epithelial cells (CFBE-Luc) were used, and constitutive bioluminescence was used to monitor viability. These cells were cultivated either under submerged conditions or in porous inserts to better model the human airway at the air–liquid interface. In the latter condition, trans-epithelial electrical resistance measurements were used to assess the formation of tight junctions in the obtained epithelial monolayer (not shown). *P. aeruginosa* was mixed with either of the two [Ru(II)] before addition to the cells. After light treatment was applied, the bacterial mixture was replaced with a fresh medium (without antibiotics) for submerged cells, or it was discarded for cells cultured in inserts.

**Figure 10:**
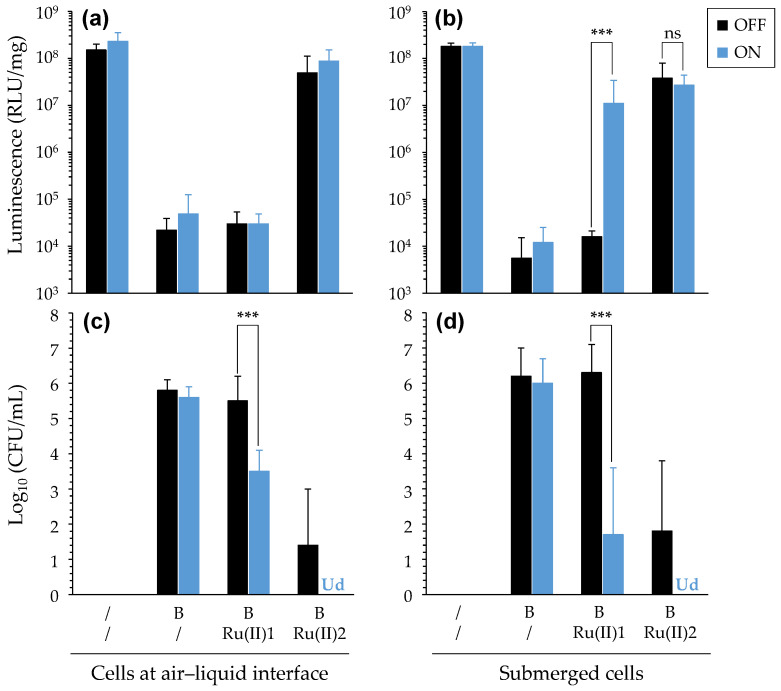
PDT effects of [Ru(II)] in the presence of eukaryotic cells cultured in different conditions. Results were obtained when assaying bacteria (B) with CFBE-Luc cells, with the latter being cultivated either at the air–liquid interface (**a**,**c**) or under submerged conditions (**b**,**d**). Luminescence measurements ((**a**,**b**); expressed in the unit of RLU/mg of total protein) and CFU enumeration (**c**,**d**) were used to assess cell viability and antibacterial effects, respectively. Mean ± SD with *n* = 3 and N = 2. ***, *p*-value ≤ 0.001; “ns”: non-significant. “Ud”: undetectable.

After incubation for 24 h, irrespective of the light treatment applied, bacteria without [Ru(II)] efficiently grew in both cell culture conditions, which resulted in a strongly reduced bioluminescence indicative of cell injuries/death due to the growing bacteria (Figure 10). When assaying [Ru(II)], quite similar results were obtained with submerged cells and cells cultured at the air–liquid interface. With [Ru(II)]1, no dark toxicity towards bacteria was noticed, whereas the bacterial load was significantly reduced upon light irradiation. However, this effect was only partial, and bacteria were still able to grow to some extent. As a result, the cells were affected, particularly those cultured at the air–liquid interface, whereas those under submerged conditions still retained some metabolic activity (as reflected in quite-high-bioluminescence signals). When using [Ru(II)]2, it was noticeable that, although some dark toxicity towards bacteria occurred, light treatment allowed us to obtain a full eradication of bacteria. At the same time, cell viability was preserved, as demonstrated with bioluminescence levels similar to that of control non-infected cells (Figure 10). From these results, no obvious adverse effects towards CFBE-Luc attributable to [Ru(II)] were found.

## 4. Discussion

[Ru(II)] may be used in many biomedical applications, notably to fight against pathogenic micro-organisms [20]. However, to date, these compounds have been considered and tested in experimental conditions usually far from clinical settings. Furthermore, their mechanism of action, which is required for obtaining approval for a given biomedical application, is not fully unveiled yet or has been investigated only under some specific conditions. In vivo studies—or studies performed under in vivo approaching conditions—using [Ru(II)] are still lacking, particularly for antimicrobial purposes [11,49]. In the present study, we questioned whether [Ru(II)]-based aPDT could be used to fight against lung pathogens responsible for infections in CF patients, especially those in the advanced stages of the disease, for which the chronic use of multiple antibiotics and the resulting selection of MDR lung pathogens severely limits the options of antibiotic coverage [35].

CF lung disease could be eligible for treatment with aPDT for several reasons, including the following. (i) Lung airways can be targeted with a local administration method, especially via aerosol that allows one to obtain a high load of in situ active drugs with low systemic side-effects [50,51,52]. (ii) Because PDT reactions only occur in areas where the PS accumulates and light is applied, lung pathogens settling at the epithelium surface can be the first biological targets of PDT-induced oxidative stresses. (iii) Lungs are located inside the chest and are thus non-exposed to ambient light, which avoids or limits photosensitivity for the host. (iv) The residence time of inhaled drugs within the lungs determines the duration of antibacterial activity at the site of infection, which may be prolonged for metal cations due to slower pulmonary absorption compared with some antibiotics [51,53]. (v) CF airways are characterized with hypoxia, especially inside the mucus and biofilms where pathogens develop. Although O_2_ has been claimed to be a pivotal element in PDT reactions, some evidence have been obtained recently for oxygen-independent photoinactivation of bacteria with some PS [47].

Besides these eligibility criteria, CF is characterized by many pathophysiological disorders, making lung infections particularly hard to treat. Loss-of-function mutations in the CFTR impair the efflux of anions such as chloride and bicarbonate across the apical membrane of the respiratory epithelial cells. This has many consequences, considering the multiple roles of bicarbonate secretion in the airways: HCO_3_^−^ notably drives ionic content and fluid on epithelial surfaces, regulates the local pH, allows mucins to unfold and become slippery and contributes to innate immunity. Slippery mucins are needed to trap microorganisms and transport them away from epithelial surfaces, whereas a neutral pH is needed for optimal bacterial killing by antimicrobial proteins such as defensins [54]. When CFTR is dysfunctional, the lack of bicarbonate secretion disrupts these normal processes. Electrolytic and osmolality imbalance results in the accumulation of highly viscous secretion, leading to recurrent infections and chronic colonization by opportunistic pathogens. Particularly, *P. aeruginosa* in the lungs of CF patients can grow to high densities in the stagnant mucopurulent secretions that are depleted in oxygen [55]. All these disturbances can impact the efficiency of antimicrobials [30]. Before this study, it was unclear whether [Ru(II)] could exert some aPDT under CF lung micro-environments.

In a previous study, 17 mononuclear, homo- or heteroleptic, ruthenium(II) polypyridyl complexes were evaluated regarding their ability to photo-inactivate bacterial clinical isolates [23]. This screening was carried out under standard in vitro conditions, using planktonic bacteria in phosphate-buffered saline (PBS) at a pH of 7.4. This allowed us to highlight the potent aPDT effects of some [Ru(II)] on CF-associated *P. aeruginosa* isolates. Among the compounds investigated, the highly hydrophilic [Ru(Phen)_3_]^2+^ (PF_6_^−^)_2_ showed the highest ^1^O_2_ quantum yield, but, likely due to a defect to stand in the near environment of bacteria, it was almost ineffective in the experimental condition considered. Contrastingly, the much less efficient ^1^O_2_ producer [Ru(Phen)_2_(Phen-T-Fluorenyl)]^2+^ (PF_6_^−^)_2_ was highlighted as the most effective in that study. We speculated that this compound reached a compromise with respect to lipophilicity, which was found to negatively impact ^1^O_2_ generation but was required to efficiently interact with bacteria. Although these [Ru(II)] exhibited a common core, the engraftment of a (Phen-T-Fluorenyl) ligand was thus shown to strongly impact the photophysical and physicochemical properties determining photodynamic antibacterial effects.

To expand this work further, we here focused on the two above-mentioned [Ru(II)], herein noted as [Ru(II)]1 and [Ru(II)]2, respectively (Figure 1), to learn more details about bacterial photo-inactivation under a wider variety of experimental conditions than considered before [23] and better approaching CF lung disorders (Table S1). We first conceived and qualified a custom-built robust experimental system allowing us to perform multiple tests under various well-controlled experimental conditions (Figure 2). The protocol used proved to be reproducible and efficient as a result of both the number of tests performed in parallel and the requirement of only a few (repeated twice) and short-lived (5 to 15 min each) successive irradiations. Considering the lower hydrophilicity of [Ru(II)]2, it was formulated with chlorides instead of hexafluorophosphates to increase water solubility. A stepwise complexified approach was followed to investigate the antibacterial photodynamic effect of [Ru(II)] when varying parameters related to environmental conditions.

(1) The photophysical characterizations of [Ru(II)] showed very different behaviors for the two [Ru(II)], likely primarily associated with their different water-solubilities. In particular, [Ru(II)]1 absorption in the UV-visible spectrum was almost the same in water and in saline, whereas [Ru(II]2 absorption was disturbed in NaCl in a saline-dose-dependent manner. However, for NaCl concentrations in the range of that typically found in CF airways [56], the UV-vis spectrum of each compound was similar to that in water (Figure 3). Different abilities for ^1^O_2_ and intracellular ROS generations were measured (Figure 4), further detailing the results previously obtained [23]. The assays conducted to study the interaction of [Ru(II)] with bacteria possibly leading to some membrane permeabilization allowed us to obtain insight into the potential mechanism(s) of action involved (Figure 5, Figures S3 and S4). Collectively, these assays underpinned the main role of osmotic strength and the composition of the surrounding medium determining, in different ways, the ability of [Ru(II)] to display photodynamic properties.

(2) The impact of different parameters on aPDT during illumination was then assessed, including salinity (Table 1), pH (Figure 6) and oxygen availability (Figure 7). In many cases, contrastive results were obtained with the two [Ru(II)] evaluated. PDT towards bacteria in ASM could also be measured, provided that the light treatment was adapted, underscoring the impact of complex medium compositions and the need for sufficient light diffusion to activate [Ru(II)] (Figure 8). These results are consistent with findings reported earlier with other PS [57].

(3) Bacteria grown in different conditions were finally evaluated to better take into account CF lung infectious conditions. The latter is a complex lifelong evolving polymicrobial disease, typically characterized with “a switching” between *S. aureus* (early colonizer) and *P. aeruginosa* (predominant in adulthood), which constitutes a turning point in the disease [58]. Particularly, *P. aeruginosa* adopts a biofilm lifestyle that allows this pathogen to elude antimicrobials while reaching high titers. (i) Here, we demonstrated the possibility to use [Ru(II)] in photoinactivation of a given bacteria, whether it was cultivated alone or mixed with another species (Figure S9). (ii) Biofilm eradication assays were also carried out, according to two experimental set-ups, which we named pre-delivery and post-delivery methods, depending on the timing of the addition of [Ru(II)] to bacteria with respect to the biofilm formation. In independent experiments, some obvious effects were obtained showing antibiofilm effects of [Ru(II)] either entrapped in or even deposited at the surfaces of biofilms. Remarkably, upon light irradiation, “pre-delivered” [Ru(II)]1 strongly reduced the bacterial load in *P. aeruginosa* biofilm, as efficiently as an antibiotic used as a positive control (Figure 9). Following the post-delivery method, some PDT effect on the metabolism of bacteria in biofilms was demonstrated (Figure S11), which could be associated with some variable reductions in the bacterial load in biofilms (not shown); however, in every case, no modification of the total biomass could be found (not shown). The ability of [Ru(II)] to exert some aPDT effect even from the surfaces of biofilms suggests that the diffusion of ROS was not prevented by the biofilm thickness. These results are actually in accordance with observations reported before with other PS [59]. However, it is obvious that weaker effects were obtained when assaying compounds following the post-delivery method; this clearly points out the limited ability of [Ru(II)] to diffuse and get close to the target bacteria in biofilms, due at least in part to the biofilm composition and the high amount of slim especially produced by PA19660. If this limitation is circumvented (such as experimentally performed in this study; Figure 9), [Ru(II)] can exert potent PDT effects against biofilm-growing bacteria, despite the presence of extracellular polymeric substances, and towards bacteria at different growth stages. It is noteworthy here that complementary assays indeed showed the potential of [Ru(II)] to act on bacteria growing in either stationary or exponential phases (not shown). Besides this, the demonstration of aPDT from biofilm-embedded [Ru(II)] is consistent with the demonstration of the ability of [Ru(II)] to retain some activity in less oxygenated conditions (Figure 7). In light of the above, we can speculate that [Ru(II)] may display PDT effects through different photochemical pathways, including an oxygen-independent mechanism [47]. This warrants further investigations to determine whether [Ru(II)] can actually retain their activity under the almost-anaerobic conditions that can be found in CF airways [60]. (iii) Last, we highlighted the potential of [Ru(II)] towards bacteria grown in the presence of eukaryotic cells. Oxidative stress targeting bacteria could be obtained while preserving underlying cell cultures either in submerged conditions or at the air–liquid interface (Figure 10).

All combined, the results obtained gauge the potential of [Ru(II)] under specific conditions approaching CF lung infections. This study further confirmed the potential of [Ru(II)]2, as shown in our previous study that used less optimized illumination systems [23]. Furthermore, salinity is clearly identified as a main limitation for [Ru(II)]-based PDT. However, we herein demonstrate that modulating the experimental conditions can drastically modify the ability of a given [Ru(II)] to exert aPDT; an ineffective compound could indeed become effective under adapted experimental conditions allowing to overcome its limitations. However, it is noteworthy that, depending on the various conditions evaluated, one or the other [Ru(II)] performed more efficiently.

Following the present study, several optimizations can be proposed addressing the chemical structure and/or the formulation of the bare compounds evaluated herein. It is indeed obvious that the [Ru(II)] considered may be optimized or replaced by other candidates, and from this point of view, there is ample room for improvement. The results obtained with [Ru(II)] suggest that they may be highly efficient, provided their respective drawbacks are bypassed, which may be done following different strategies. For instance, PEGylated derivatives of [Ru(II)] may be less prone to aggregation and precipitation while showing improved mucus penetration and antimicrobial activity against bacterial biofilms, as recently reported, for instance, for tobramycin [61]. Derivatives triggered with two–photon absorption in near–infrared (NIR) or IR could allow for a better penetration of the excited beam [24,25,62], which may be crucial for targeting infections in deep organs, such as the lungs. The encapsulation of ruthenium complexes has also been reported as a means to overcome the limitations of PS in biological fluids [63,64]. By providing additional specificity for the bacteria to eliminate, these strategies may allow for avoiding/reducing adverse events in CF patients in which a sub-optimal antioxidant protection was reported as a cause of pre-existing oxidative stress [65].

The study design used herein allowed highlighting the impact of multiple parameters typical of CF lung pathophysiology, especially increased osmotic strength, acidic pH, lower oxygen availability, mucus secretion and biofilm formation. Other parameters should be considered, such as lung surfactant, as evaluated with other PS [66]. Some technical optimizations are anticipated in follow-up studies for better assessing the actual feasibility to perform efficient aPDT in CF lung airways. The optimization of light treatment is crucial, as suggested with prolonged illuminations that herein allowed us to achieve better results when assaying complex samples such as ASM and biofilms. In line with a complexification by the incremental implementation of multiple barriers, it can also be informative to perform assays using ALI cultures of primary human bronchial epithelial cells, producing mucus that could be colonized with biofilm-forming lung pathogens, thus better recapitulating CF lung disease [67]. This integrated approach should be used before eventual evaluations conducted in vivo in animals, in accordance with the 3R rule in animal experimentation. Although investigations using CF animal models can provide additional clues, it is noteworthy that currently available in vivo models are still not fully satisfying, since they fail to recapitulate the disease, as observed in CF humans. Notably, the lung pathology is milder in mouse models compared to humans, and CF mice do not spontaneously develop airway infections [68]. Thus, studies performed under in vitro experimental conditions remain crucial.

## 5. Conclusions

This study aimed to provide proof of principle for a potential therapeutic use of [Ru(II)]-based aPDT in CF lung airways. Beyond the obvious importance of the molecular engineering of [Ru(II)], this work corroborates that multiple conditions must be met to reach optimal activities. We indeed point out that microbial photo-inactivation can be impacted by environmental features characteristic of the pathophysiological context of CF lung airways. Accordingly, this study may be used to define requirement specifications and guidelines for aPDT in this specific biological context and to design dedicated [Ru(II)] derivatives. In addition, the use of an appropriate administration route and method to ensure safe and efficient in situ light activation is required. Intensive works are currently ongoing on these topics in our laboratories. In conclusion, metal complexes such as [Ru(II)] constitute credible candidates for combating lung infections as a result of their unique photodynamic properties and also other modes of action, including redox activation, ligand exchange and the depletion of substrates involved in cellular processes [14,15,69]. Further research will allow for the gaining of breadth and depth in the utility and feasibility of [Ru(II)] for application in CF lung airways.

## Figures and Tables

**Table 1:** MIC ^1^ of [Ru(II)] following light treatment performed either in water or in 1X NaCl.

			[Ru(II)]1		[Ru(II)]2	
			OFF	ON	OFF	ON
*S. aureus*	RN4220	NaCl	>64 ^2^	>64	64	32
		Water	>64	32	64	8
	N315	NaCl	>64	>64	64	64
		Water	>64	64	64	32
*P. aeruginosa*	PA19660	NaCl	>64	64	>64	32
		Water	8	8	8	8
	PAH	NaCl	>64	>64	8	8
		Water	>64	64	Nd	Nd

^1^ The concentrations evaluated were: 2, 8, 32 and 64 µM; ^2^ in µM. Representative data obtained with *n* = 3 and N = 2. “Nd”: not determined.

## Data Availability

The raw/processed data required to reproduce these findings are either available from the supporting materials or cannot be shared at this time, as the data also form a part of ongoing studies.

## Supplementary Materials

The following supporting information can be downloaded at: https://www.mdpi.com/article/10.3390/pharmaceutics14081664/s1, Table S1: Patho-physiological disorders typically found in lung airways of CF patients that could interfere with aPDT; Table S2: Bacteria used and their characteristics; Table S3: Volumes of acetic acid and sodium acetate stock solutions to mix for obtaining buffers at desired pH; Figure S1: Further characterizations of the illumination set-up; Figure S2: Impact of light treatment on UV-visible spectra of [Ru(II)] in water or in saline solutions; Figure S3: Production of singlet oxygen upon light treatment of [Ru(II)] in different conditions; Figure S4: Interaction assay between [Ru(II)] and bacteria when mixed either in water or in 1X NaCl; Figure S5: [Ru(II)] effects on cell surface integrity of bacteria; Figure S6: Growth kinetics of bacteria following transient incubation in acetate buffers at various pH; Figure S7: Assessment of hypoxic conditions with oil deposition at the surface of the medium; Figure S8: PDT effects of [Ru(II)] in ASM; Figure S9: PDT effects of [Ru(II)] on *S. aureus* when cultured alone (A) or in mixtures (M) with *P. aeruginosa*; Figure S10: Biomass quantification via crystal violet (CV) staining; Figure S11: PDT effects of [Ru(II)] towards bacteria in biofilms (“post-delivery antibiofilm assay”).

## Abbreviations

ALI, air–liquid interface; aPDT, antimicrobial photodynamic therapy; ASL, airway surface liquid; ASM, artificial sputum medium; CF, cystic fibrosis; CFBE-Luc, bioluminescent cystic fibrosis bronchial epithelial cell line; CFU, colony forming unit; DCFH-DA, 2′,7′-dichlorofluorescin diacetate; JGB, janus green B; LED, light emitting diode; MDR, multidrug-resistant; MRSA, methicillin-resistant *S. aureus*; OFF, non-illuminated; ON, subjected to light treatment; Phen, 1,10-phenanthroline; PI, propidium iodide; PS, photosensitizer; RFU, relative fluorescence unit; RLU, relative luminescence unit; ROS, reactive oxygen species; RT, room temperature; [Ru(II)], ruthenium(II) polypyridyl complexes; SOSG, singlet oxygen sensor green; TEER, trans-epithelial electrical resistance; ^1^O_2_, singlet oxygen.
