# Supplementary material for: Ruthenium(II) Polypyridyl Complexes for Antimicrobial Photodynamic Therapy: Prospects for Application in Cystic Fibrosis Lung Airways
Source: Pharmaceutics. 2022 Aug 10;14(8):1664. doi: 10.3390/pharmaceutics14081664 (PMC9412327; doi:10.3390/pharmaceutics14081664)
Supplement: Supplementary file 1 [file pharmaceutics-14-01664-s001.zip › pharmaceutics-1822461-supplementary.pdf]

pharmaceutics

Ruthenium(II) Polypyridyl Complexes for Antimicrobial Pho-todynamic Therapy: Prospects for Application in Cystic Fibrosis Lung Airways

Raphaëlle Youf, Adeel Nasir, Mareike Müller, Franck Thétiot, Tanguy Haute, Rosy Ghanem, Ulrich Jonas, Holger Schönherr, Gilles Lemercier, Tristan Montier and Tony Le Gall

Table of contents

1. Supplementary Introduction ... 2
2. Supplementary Materials and Methods ... 2
2.1. Illumination system ... 2
2.2. Bacteria ... 3
2.3. Preparation of acetate buffers ... 3
2.4. Surface charge measurements ... 3
2.5. Polymicrobial assay ... 3
2.6. Establishment of the CFBE-Luc cell line ... 3
2.7. Resazurin assay ... 4
3. Supplementary Results ... 4
List of abbreviations ... 9
References ... 10

1. Supplementary Introduction

Table S1. Patho-physiological disorders typically found in lung airways of CF patients that could interfere with aPDT.

Feature	Situation in non-CF individuals versus in CF patients		Ref
ASL salinity	Non-CF individual	$[Na^+]$=50 mM, $[Cl^-]$=37 mM	[1]
	CF patient	$[Na^+]$=90 mM, $[Cl^-]$=85 mM	
ASL pH	Non-CF individual	neutral (~ 7.35 ± 0.05)	[2]
	CF patient	acidic (~ 6.70 ± 0.03)	
Mucus	Non-CF individual	normal mucus production; effective mucociliary clearance	[3]
	CF patient	sticky and viscous mucus; ineffective mucociliary clearance	
Oxygenation	Non-CF individual	185 mM dissolved oxygen; FEV_1>70%	[4]
	CF patient	≤ 3 μM dissolved oxygen in mucus; FEV_1<70%	[5]

"FEV_1": forced expiratory volume in 1 second.

2. Supplementary Materials and Methods

2.1. Illumination system

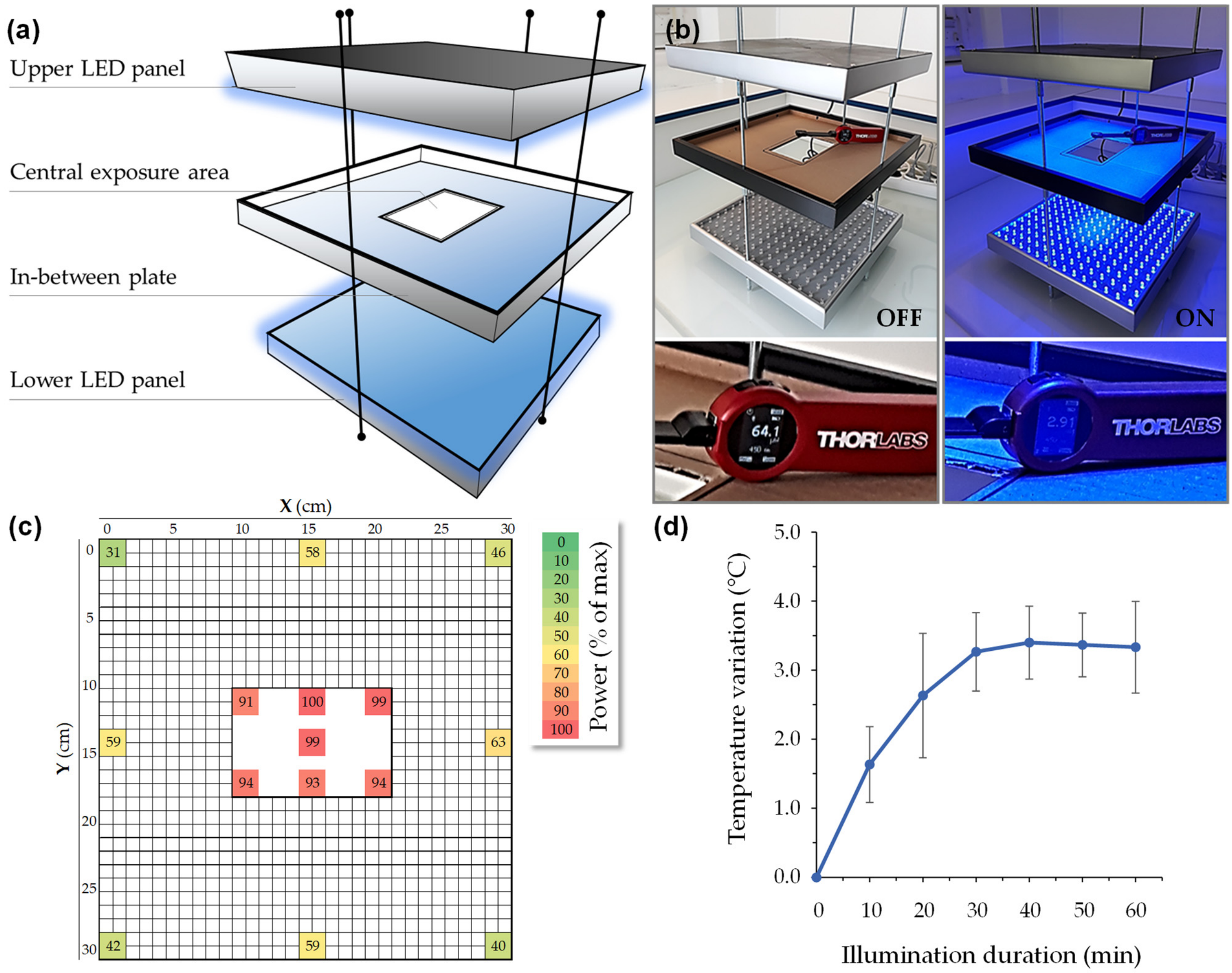

Figure S1. Further characterizations of the illumination set-up. Schematic representation (**a**) and pictures (**b**) of the setup showing LED turned OFF (left) or ON (right) and the power meter used to measure the light emitted. Assessment of the light received in different locations of the in-between plate, in the empty area and on the borders (**c**). Values are expressed as a percent of the maximal power measured (100%). Temperature variation measured in the central (empty) area during 1 h of continuous illumination (**d**). Mean ± SD, with n = 3.

2.2. Bacteria

Table S2. Bacteria used and their characteristics

Species	Strain	Origin/characteristics	ATB [1]	Ref.
S. aureus	RN4220	Laboratory strain generated through UV and chemical mutagenesis of *S. aureus* strain NCTC 8325-4.	0/19	[6]
S. aureus	N315	MRSA strain isolated from a pharyngeal smear of a Japanese patient in 1982.	8/19	[7]
P. aeruginosa	PAH	MDR strain isolated from a sputum of a CF patient in Brest hospital in 2009.	14/17	[8]
P. aeruginosa	PA19660	*P. aeruginosa* (Schroeter) Migula (ATCC 19660), originally isolated from septicemia in a patient from Peru, Lima.	Nd	ATCC

[1] Number of antibiotic resistances; detailed antibiotic resistance profile was published earlier [8]; "Nd": not determined.

2.3. Preparation of acetate buffers

A stock solution of 0.2 M acetic acid was prepared by mixing 575 µL of glacial acetic acid with 49.425 mL of sterile water. A stock solution of 0.2 M sodium acetate was prepared by mixing 1.36 g sodium acetate tri-hydrate in 50 mL of sterile water. Both solutions were sterilized by filtration through a 0.2 µm filter. Then, 5 mL of acetate buffer solutions at the desired pH were obtained as detailed in Table S3.

Table S3. Volumes of acetic acid and sodium acetate stock solutions to mix for obtaining buffers at desired pH.

Buffer pH	0.2 M acetic acid V (mL)	0.2 M sodium acetate V (mL)
6.0	0.200	4.800
6.5	0.100	4.900
7.0	0.020	4.980

2.4. Surface charge measurements

Zeta potential measurements were performed using a Nano ZS Zetasizer (Malvern Instruments, Palaiseau, France). Following centrifugation, bacterial pellets were suspended in 1 mL of sterile deionized water and were then introduced in a folded capillary DTS1070 cell (Malvern Instruments, Palaiseau, France). For each sample, the device was programmed to perform 5 successive measurements, each repeated at least 10 times, as recommended by the manufacturer.

2.5. Polymicrobial assay

Bacteria (either *S. aureus* RN4220 or *P. aeruginosa* PA19660) were first grown overnight in LB broth. They were then harvested by centrifugation and were then resuspended in LB to prepare four types of samples: two "mono-cultures" (RN4220 or PA91660 alone) and two "co-cultures" (mixtures of RN4220 and PA19660, which were assayed either immediately after mixture or following an additional incubation for 4 h). In every case, the bacterial density of each of the bacteria was adjusted to 10^5 CFU/mL before PDT treatment. The latter was performed by mixing the bacteria with [Ru(II)] (to reach 50 µM [Ru(II)]1 or 0.2 µM [Ru(II)]2). After the light treatment was applied, serial dilutions (1:10) were performed and spread on selective media, i.e. Mannitol Salt Agar (Thermo Fischer Scientific, Hampshire, UK) for *S. aureus* and Pseudomonas Isolation Agar (Sigma Aldrich, St. Louis, MO, USA) for *P. aeruginosa*, to determine the CFU of each bacterial species in every condition.

2.6. Establishment of the CFBE-Luc cell line

A total of 24 h before transduction, CFBE cells were seeded into a 24-well plate, using 100,000 cells in 800 µL of EMEMc per well. The cells were transduced using lentiviral particles containing a luciferase transgene. The estimated amount was 10 particles per cell or 1 million particles per well. The culture medium was

changed 24 h after transduction. When the cells reached confluence, they were transferred to a 6-well plate to proceed with clonal selection using an 8x8 mm cloning cylinder. This allowed us to select a limited number of cells (typically 1 to 2 cells) that were transferred to a 96-well plate. Following the clonal selection step, cells were allowed to proliferate until they reached a sufficient quantity to check the integration of the transgene and for cryopreservation. To confirm transgene integration and functionality, a polymerase chain reaction specific for the transgene was carried out using forward (5′-ACACCCGAGGGGGATGATAA-3′) and reverse (5′-GGCGACGTAATCCACGATCT-3′) primers, and luminescence was quantified using the Luciferase Assay System (Promega). Parental cells were used as controls.

2.7. Resazurin assay

The metabolic state of bacteria in biofilm was determined through a resazurin cell viability assay, as described before [9,10]. Briefly, biofilms were first washed twice with saline solution before incubating with 1X PBS solution containing 10% (V/V) of resazurin dye (Canvax, Córdoba, Spain). After 24 h incubation at 37°C, the absorbance was measured at 570 nm. According to the manufacturer, the signal generated from the assay is proportional to the number of living cells in the sample. The absorbance determined with bacteria in untreated biofilm was used to set 100% biofilm viability.

3. Supplementary Results

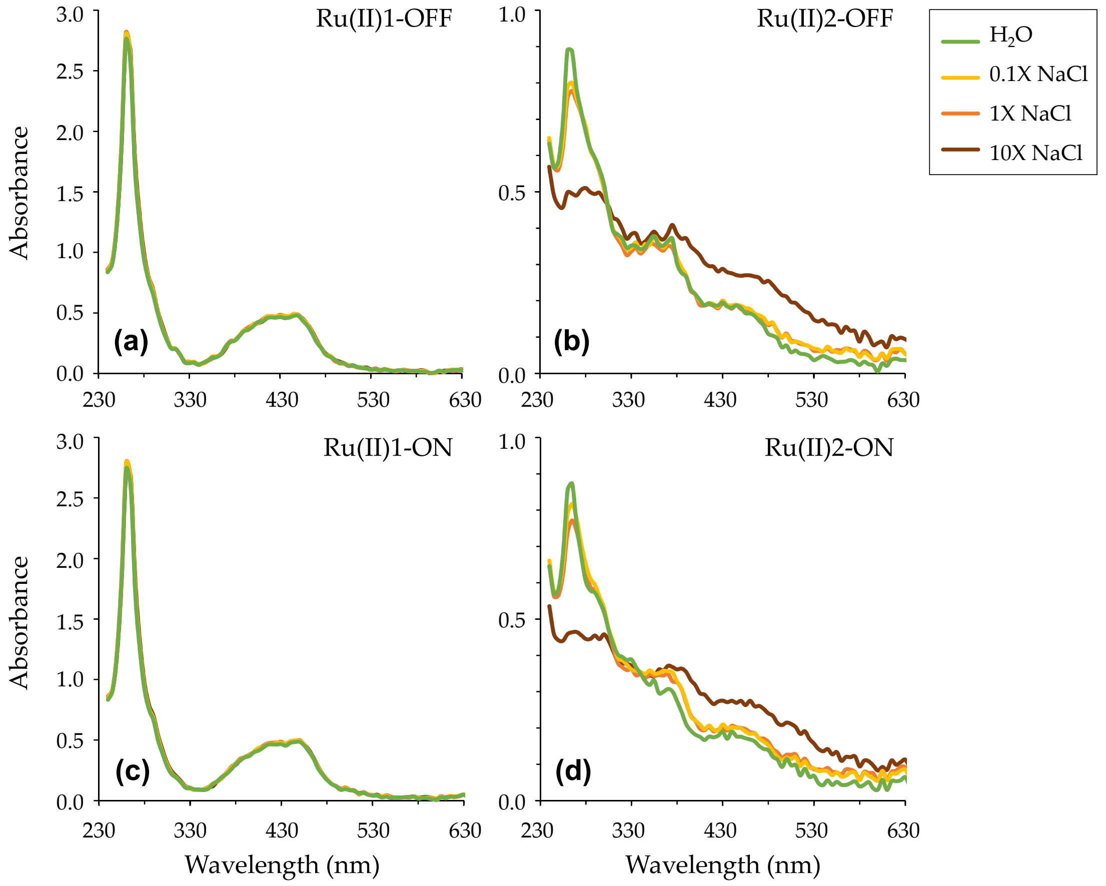

Figure S2. Impact of light treatment on UV-visible spectra of [Ru(II)] in water or in saline solutions. [Ru(II)]1 (**a**, **c**) and [Ru(II)]2 (**b**, **d**) absorption in the UV-visible spectrum was determined before (**a**, **b**) and then after (**c**, **d**) light treatment. In every test, [Ru(II)] concentration was 50 µM in 200 µL. Mean ± SD with $n = 3$ (representative results of N = 3).

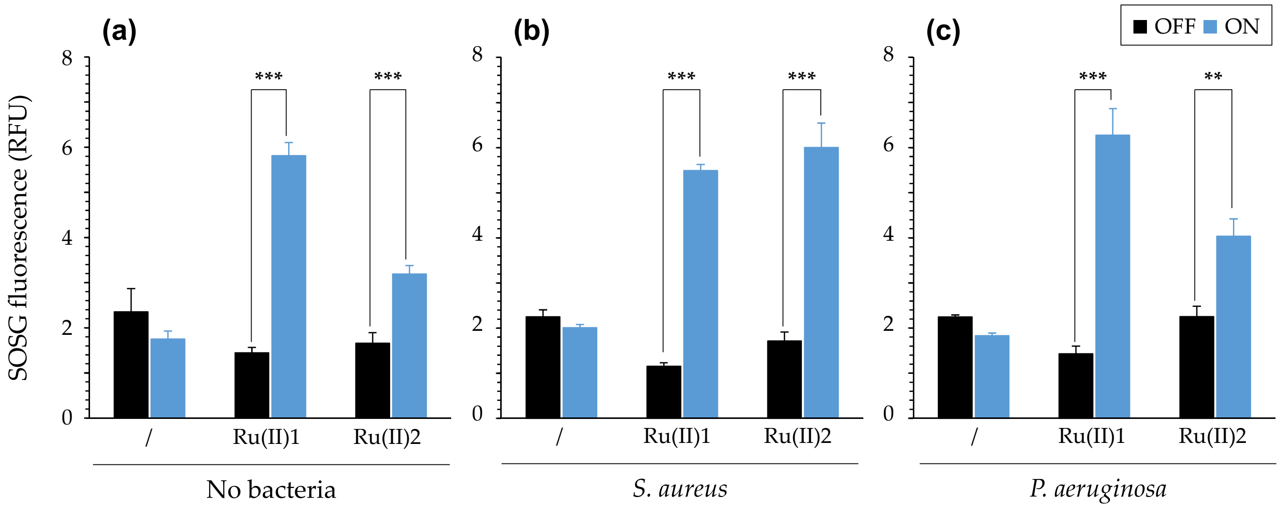

Figure S3. Production of singlet oxygen upon light treatment of [Ru(II)] in different conditions. Measurements were performed in the absence (**a**) or in the presence of bacteria, with either *S. aureus* RN4220 (**b**) or *P. aeruginosa* PA19660 (**c**). [Ru(II)] = 25 µM in Tris-HCl. Mean ± SD with $n = 3$ (representative results of N = 2). ***, p-value ≤ 0.001; **, p-value ≤ 0.01.

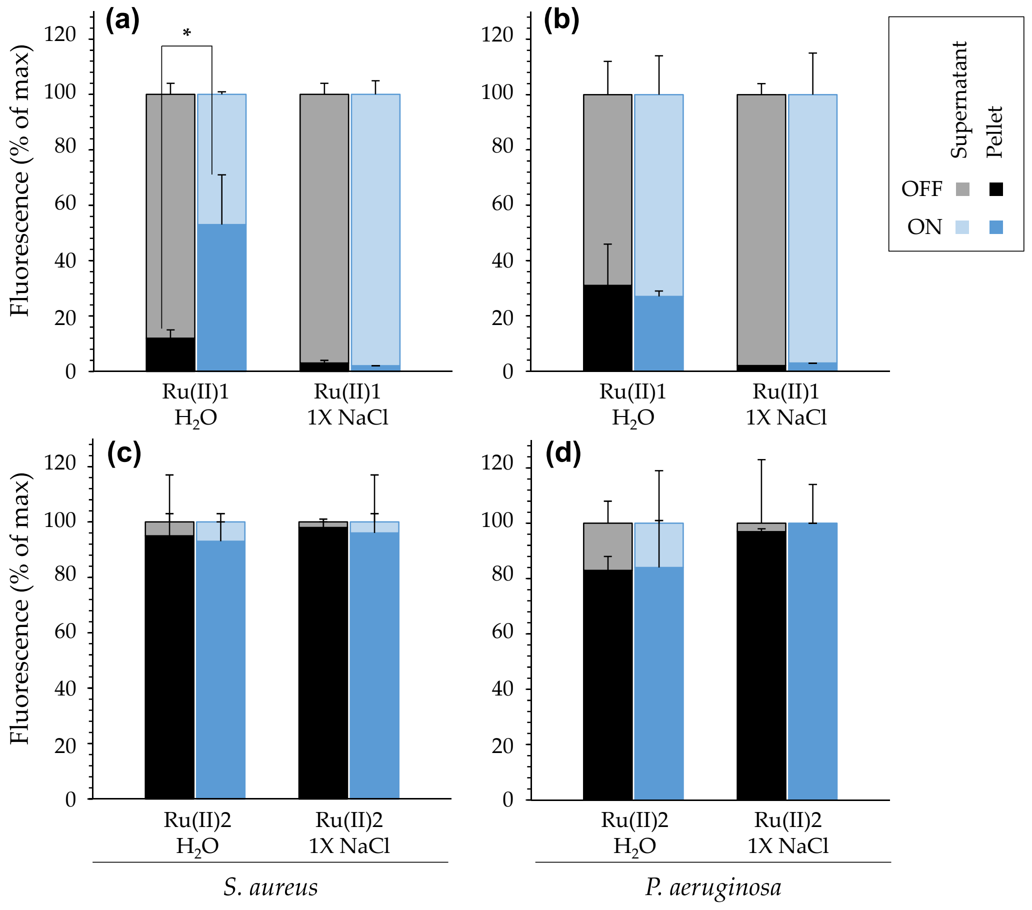

Figure S4. Interaction assay between [Ru(II)] and bacteria when mixed either in water or in 1X NaCl. Results were obtained using [Ru(II)]1 (**a**, **b**) or [Ru(II)]2 (**c**, **d**) with *S. aureus* N315 (**a**, **c**) and *P. aeruginosa* PAH (**b**, **d**). [Ru(II)] = 25 µM. Mean ± SD with $n = 3$ and N ≥ 2. *, p-value ≤ 0.05.

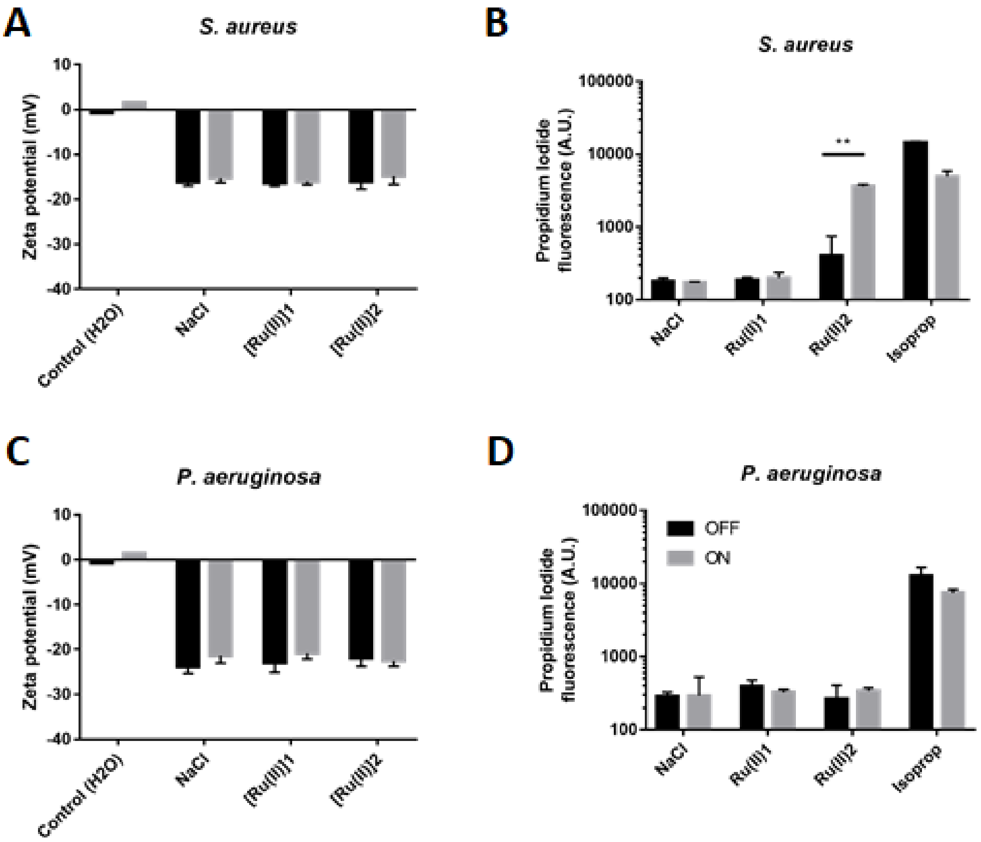

Figure S5. [Ru(II)] effects on cell surface integrity of bacteria. Zeta potential and permeability to propidium iodide of *S. aureus* RN4220 and *P. aeruginosa* PA19660 were assessed before and immediately after light treatment. Mean ± SD with $n = 3$ and N = 2. **, p-value ≤ 0.01.

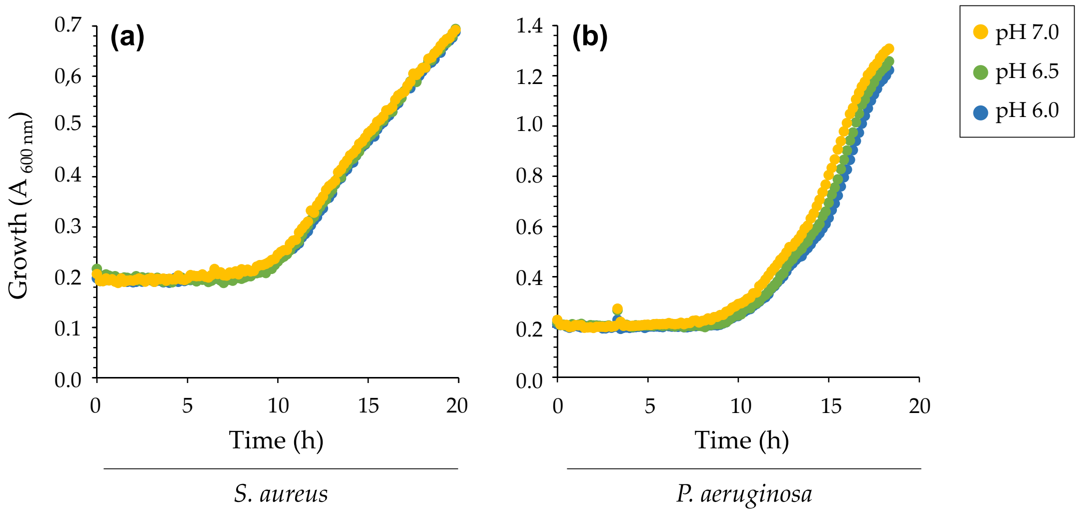

Figure S6. Growth kinetics of bacteria following transient incubation in acetate buffers at various pH. Results were obtained with *S. aureus* RN4220 (**a**) and *P. aeruginosa* PA19660 (**b**). Mean ± SD with $n = 3$ and N = 1.

We evaluated the possibility to achieve reduced oxygenation in solution. For this purpose, the deposition of sunflower oil (showing neither an antibacterial effect nor an effect on the growth of any bacteria evaluated) at the surface of the latter was used to prevent any contact with the ambient oxygenated atmosphere. To confirm the achievement of an anaerobic environment, Janus Green B (JGB) was used as an oxygen indicator, as previously reported [11]. As shown in Figure S7, a strong fluorescence increase could be already measured 15 min after the deposition of oil at the surface of saline containing both JGB and bacteria. By contrast, in the absence of oil, strongly lower fluorescence was measured. Any component alone (JGB, oil, bacteria and [Ru(II)]) did not emit any significant fluorescence. Of note, neither light treatment nor the presence or absence of bacteria changed the fluorescence emitted with JGB in any case. These results thus underpin that oil deposition allowed us to achieve a hypoxic environment that could be used to assay oxygen depletion towards aPDT using [Ru(II)].

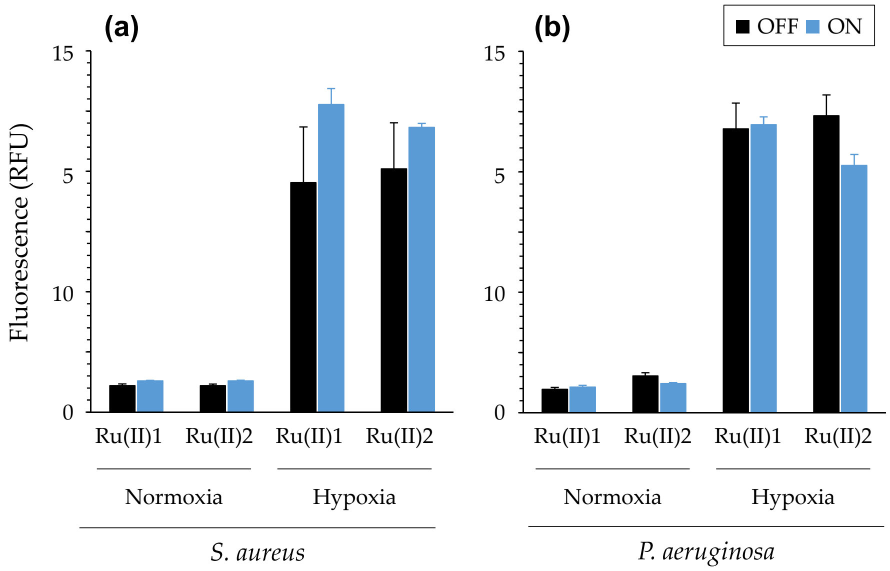

Figure S7. Assessment of hypoxic condition with oil deposition at the surface of the medium. Fluorescence was measured with excitation at 340 ± 6 nm and emission at 400 ± 22 nm. Results were obtained with *S. aureus* RN4220 (**a**) and *P. aeruginosa* PA19660 (**b**). Mean ± SD with $n = 3$ (representative results of N = 4).

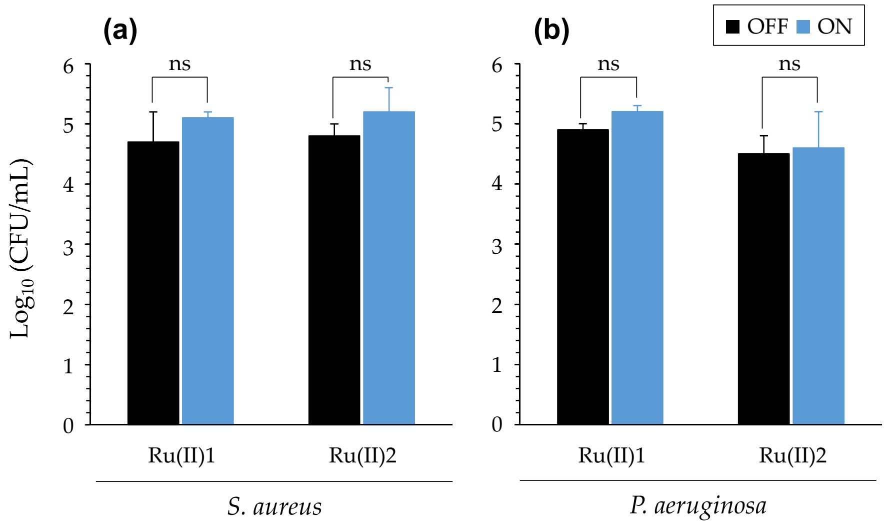

Figure S8. PDT effects of [Ru(II)] in ASM. Results were obtained with *S. aureus* RN4220 (**a**) and *P. aeruginosa* PA19660 (**b**), when using standard light treatment described in the Materials and Methods section. [Ru(II)] concentration = 25 µM in 1X ASM. Mean ± SD with $n = 3$ and N = 2. “ns”: non-significant.

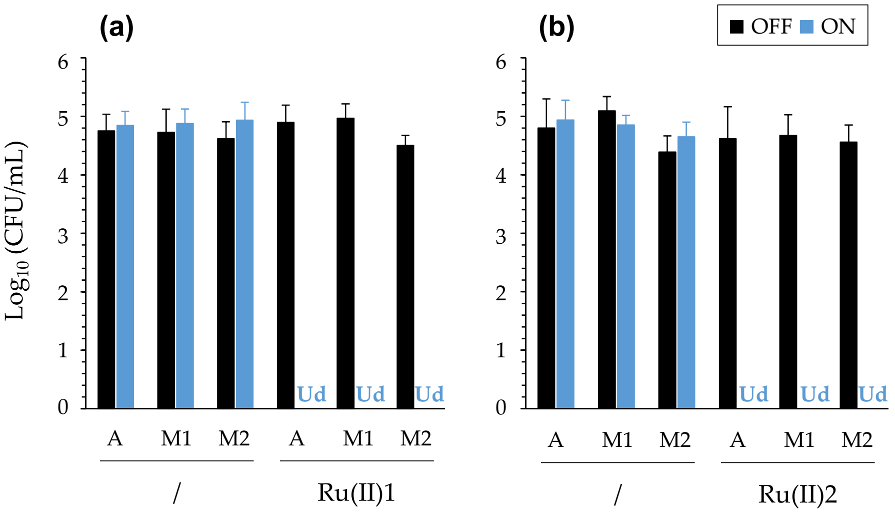

Figure S9. PDT effects of [Ru(II)] on *S. aureus* when cultured alone (A) or in a mixture (M) with *P. aeruginosa*. M1 indicates a polymicrobial preparation performed by equally mixing, just before the experiment, cultures of *S. aureus* RN4220 and *P. aeruginosa* PAH. M2 corresponds to a polymicrobial culture of *S. aureus* RN4220 and *P. aeruginosa* PAH growing together for 4 h. [Ru(II)]1 concentration = 50 µM, [Ru(II)]2 concentration = 0.2 µM in 0.5X NaCl. Mean ± SD with *n* = 3 and N = 2. "Ud": undetectable bacteria.

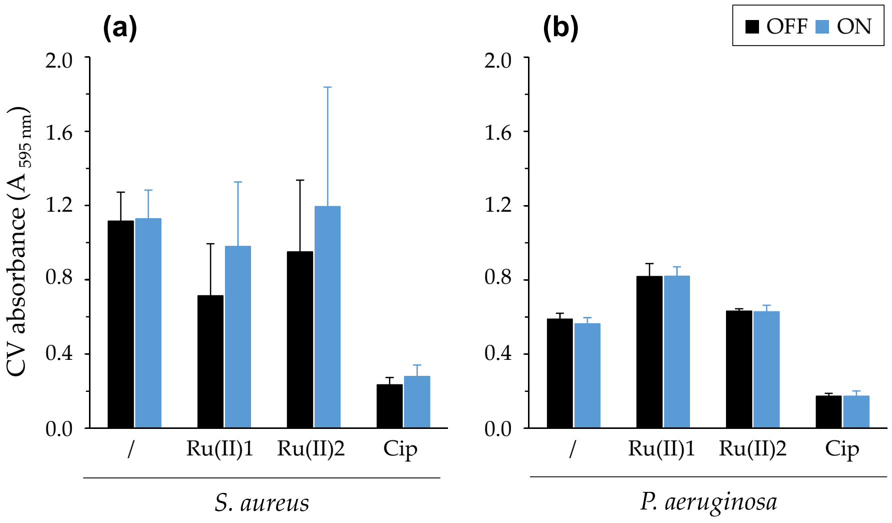

Figure S10. Biomass quantification via crystal violet (CV) staining. A595 was determined after 1/10 dilution of solubilized CV in 30% acetic acid. Results were obtained with *S. aureus* RN4220 (**a**) and *P. aeruginosa* PA19660 (**b**). "Cip": ciprofloxacin. Mean ± SD with *n* = 3 and N = 3.

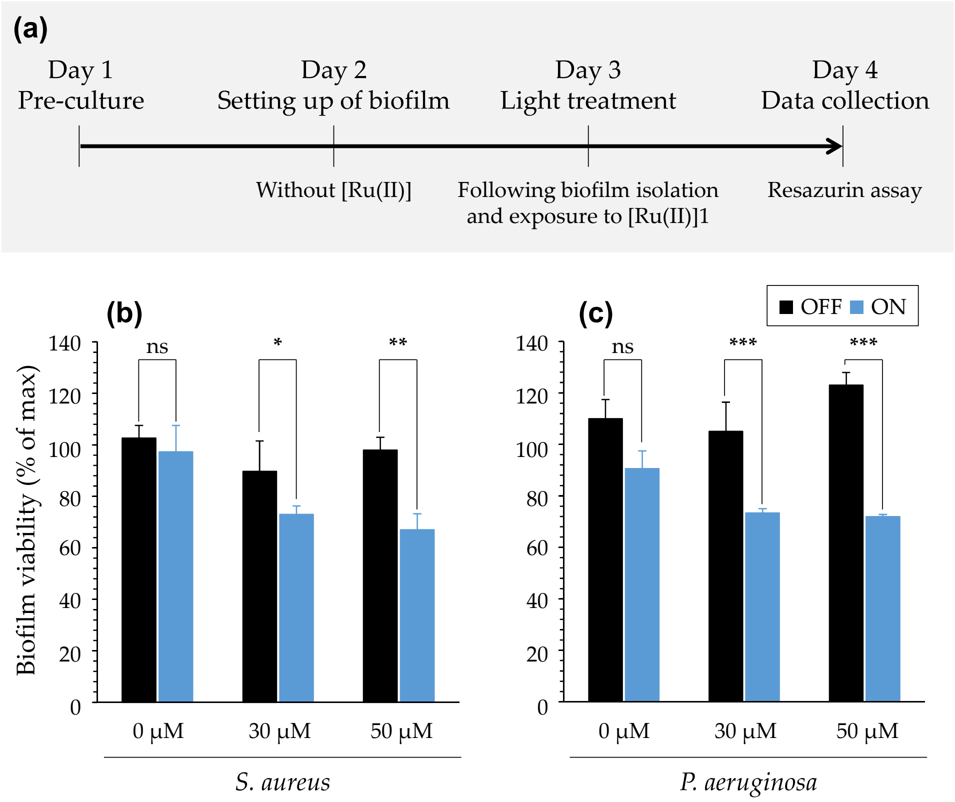

Figure S11. PDT effects of [Ru(II)] towards bacteria in biofilms ("post-delivery antibiofilm method"). The workflow of the experimentation is presented (**a**) with the results obtained using [Ru(II)]1 when assaying *S. aureus* RN4220 (**b**) or *P. aeruginosa* PA19660 (**c**). Mean ± SD with n = 2 and N = 3. ***, *p*-value ≤ 0.001; **, *p*-value ≤ 0.01; *, *p*-value ≤ 0.05; "ns": non-significant.

List of abbreviations

ALI	air–liquid interface
aPDT	antimicrobial photodynamic therapy
ASL	airway surface liquid
ASM	artificial sputum medium
CF	cystic fibrosis
CFBE-Luc	bioluminescent cystic fibrosis bronchial epithelial cell line
CFU	colony forming unit
DCFH-DA	2′,7′-dichlorofluorescin diacetate
JGB	janus green B
LED	light-emitting diode
MDR	multidrug-resistant
MRSA	methicillin-resistant *S. aureus*
OFF	non-illuminated
ON	subjected to light treatment
Phen	1,10-phenanthroline
PI	propidium iodide
PS	photosensitizer
RFU	relative fluorescence unit
RLU	relative luminescence unit
ROS	reactive oxygen species
RT	room temperature

[Ru(II)]	ruthenium(II) polypyridyl complexes
SOSG	singlet oxygen sensor green
TEER	trans-epithelial electrical resistance
$^1O_2$	singlet oxygen

References

1. Zabner, J.; Smith, J.J.; Karp, P.H.; Widdicombe, J.H.; Welsh, M.J. Loss of CFTR Chloride Channels Alters Salt Absorption by Cystic Fibrosis Airway Epithelia in Vitro. *Mol. Cell* **1998**, *2*, 397–403, doi:10.1016/s1097-2765(00)80284-1.
2. Gianotti, A.; Capurro, V.; Delpiano, L.; Mielczarek, M.; García-Valverde, M.; Carreira-Barral, I.; Ludovico, A.; Fiore, M.; Baroni, D.; Moran, O.; et al. Small Molecule Anion Carriers Correct Abnormal Airway Surface Liquid Properties in Cystic Fibrosis Airway Epithelia. *Int. J. Mol. Sci.* **2020**, *21*, E1488, doi:10.3390/ijms21041488.
3. Bustamante-Marin, X.M.; Ostrowski, L.E. Cilia and Mucociliary Clearance. *Cold Spring Harb. Perspect. Biol.* **2017**, *9*, a028241, doi:10.1101/cshperspect.a028241.
4. Alvarez-Ortega, C.; Harwood, C.S. Responses of Pseudomonas Aeruginosa to Low Oxygen Indicate That Growth in the Cystic Fibrosis Lung Is by Aerobic Respiration. *Mol. Microbiol.* **2007**, *65*, 153–165, doi:10.1111/j.1365-2958.2007.05772.x.
5. Stolz, D.; Barandun, J.; Borer, H.; Bridevaux, P.-O.; Brun, P.; Brutsche, M.; Clarenbach, C.; Eich, C.; Fiechter, R.; Frey, M.; et al. Diagnosis, Prevention and Treatment of Stable COPD and Acute Exacerbations of COPD: The Swiss Recommendations 2018. *Respir. Int. Rev. Thorac. Dis.* **2018**, *96*, 382–398, doi:10.1159/000490551.
6. Nair, D.; Memmi, G.; Hernandez, D.; Bard, J.; Beaume, M.; Gill, S.; Francois, P.; Cheung, A.L. Whole-Genome Sequencing of Staphylococcus Aureus Strain RN4220, a Key Laboratory Strain Used in Virulence Research, Identifies Mutations That Affect Not Only Virulence Factors but Also the Fitness of the Strain. *J. Bacteriol.* **2011**, *193*, 2332–2335, doi:10.1128/JB.00027-11.
7. Kuroda, M.; Ohta, T.; Uchiyama, I.; Baba, T.; Yuzawa, H.; Kobayashi, I.; Cui, L.; Oguchi, A.; Aoki, K.; Nagai, Y.; et al. Whole Genome Sequencing of Meticillin-Resistant Staphylococcus Aureus. *Lancet* **2001**, *357*, 1225–1240.
8. Le Gall, T.; Berchel, M.; Le Hir, S.; Fraix, A.; Salaün, J.Y.; Férec, C.; Lehn, P.; Jaffres, P.-A.; Montier, T. Arsonium-Containing Lipophosphoramides, Poly-Functional Nano-Carriers for Simultaneous Antibacterial Action and Eukaryotic Cell Transfection. *Adv. Healthc. Mater.* **2013**, doi:10.1002/adhm.201200478.
9. Kirchner, S.; Fothergill, J.L.; Wright, E.A.; James, C.E.; Mowat, E.; Winstanley, C. Use of Artificial Sputum Medium to Test Antibiotic Efficacy against Pseudomonas Aeruginosa in Conditions More Relevant to the Cystic Fibrosis Lung. *J. Vis. Exp. JoVE* **2012**, e3857, doi:10.3791/3857.
10. Dalecki, A.G.; Crawford, C.L.; Wolschendorf, F. Targeting Biofilm Associated Staphylococcus Aureus Using Resazurin Based Drug-Susceptibility Assay. *J. Vis. Exp. JoVE* **2016**, doi:10.3791/53925.
11. Saxena, S.; Agrawal, I.; Singh, P.; Jha, S. Portable, Low-Cost Hypoxia Chamber for Simulating Hypoxic Environments: Development, Characterization and Applications. *Med. Devices Sens.* **2020**, *3*, e10064, doi:10.1002/mds3.10064.
